# Identification of *Bradyrhizobium elkanii* USDA61 Type III Effectors Determining Symbiosis with *Vigna mungo*

**DOI:** 10.3390/genes11050474

**Published:** 2020-04-27

**Authors:** Hien P. Nguyen, Safirah T. N. Ratu, Michiko Yasuda, Neung Teaumroong, Shin Okazaki

**Affiliations:** 1 Institute of Global Innovation Research (IGIR), Tokyo University of Agriculture and Technology (TUAT), Fuchu, Tokyo 183-8538, Japan; nguyenphuochien92@gmail.com; 2 United Graduate School of Agricultural Science, TUAT, Fuchu, Tokyo 183-8509, Japan; safirahtasanervesratu@gmail.com; 3 Graduate School of Agriculture, TUAT, Fuchu, Tokyo 183-8509, Japan; ysdmichi@cc.tuat.ac.jp; 4 School of Biotechnology, Institute of Agricultural Technology, Suranaree University of Technology (SUT), Ratchasima 30000, Thailand; neung@sut.ac.th

**Keywords:** symbiosis, T3SS, effectors, NopL, *Vigna mungo*, *Bradyrhizobium elkanii*

## Abstract

*Bradyrhizobium elkanii* USDA61 possesses a functional type III secretion system (T3SS) that controls host-specific symbioses with legumes. Here, we demonstrated that *B. elkanii* T3SS is essential for the nodulation of several southern Asiatic *Vigna mungo* cultivars. Strikingly, inactivation of either Nod factor synthesis or T3SS in *B. elkanii* abolished nodulation of the *V. mungo* plants. Among the effectors, NopL was identified as a key determinant for T3SS-dependent symbiosis. Mutations of other effector genes, such as *innB*, *nopP2*, and *bel2-5*, also impacted symbiotic effectiveness, depending on host genotypes. The *nopL* deletion mutant formed no nodules on *V. mungo,* but infection thread formation was still maintained, thereby suggesting its pivotal role in nodule organogenesis. Phylogenetic analyses revealed that NopL was exclusively conserved among *Bradyrhizobium* and *Sinorhizobium* (*Ensifer*) species and showed a different phylogenetic lineage from T3SS. These findings suggest that *V. mungo* evolved a unique symbiotic signaling cascade that requires both NFs and T3Es (NopL).

## 1. Introduction

Bacteria have continuously evolved a multitude of strategies to promote infections in their hosts, including the direct injection of proteins via protein secretion systems. Numerous Gram-negative pathogenic bacteria employ a type III secretion system (T3SS) to translocate their virulence effector proteins (hereafter T3Es) directly into host cells to promote infection and pathogenesis [1]. Similarly, rhizobia, nitrogen-fixing bacteria inducing nodules on leguminous plants, have adopted such a system for symbiotic purposes [2,3,4,5,6]. Rhizobial T3SS-secreted proteins, called nodulation outer proteins (Nops), are injected into host cells as T3Es depending on an extracellular secretion apparatus [7]. Once translocated, some T3Es modulate host functions toward promoting infection and symbiosis with legumes [6], while several T3Es may be recognized as asymbiotic factors directly or indirectly via host resistance (R) proteins or specific receptors, consequently triggering immune responses that restrict nodulation [7].

*Bradyrhizobium elkanii* USDA61 (hereafter USDA61), first isolated from the soybean [8], possesses a functional T3SS controlling host-specific symbiosis with legumes [9]. Among the T3SS-secreted proteins, at least eight, including NopA, NopB, NopC, NopX, NopL, NopP, Bel2-5, and InnB, have been confirmed by extracellular protein and immunodetection analyses [9,10,11]. Three of them (NopA, B, and X) are required for T3SS components, protein secretions, and translocations [12]. In contrast, NopC, NopL, NopP, InnB, and Bel2-5 are characterized among rhizobial strains as functioning T3Es in plant cells [9,10,13]. Our previous studies demonstrate that InnB is a novel rhizobium-specific effector that functions negatively or positively in symbioses with *Vigna radiata* and *Vigna mungo*, respectively. However, the mutation of *innB* resulted in symbiotic phenotypes significantly different to those of the T3SS-deficient mutant [9]. The *innB* mutant induced fewer nodules and less plant biomass on *V. mungo* cv. PI173934 than USDA61 did, whereas the inactivation of T3SS more fully abolished nodulation [9]. However, symbiotic implications of *B. elkanii* T3SS among *V. mungo* cultivars and other *Vigna* species remain unclear. In this study, we further characterized the roles of bradyrhizobial T3SS as a determinant for symbiosis with *V. mungo* cultivars. Our results revealed that *B. elkanii* T3SS is essential for nodulating at least two South Asiatic *V. mungo* cultivars. Furthermore, T3SS-triggered symbiosis relied on a T3E cocktail, in which NopL served as the key player required for establishing early infections and nodule organogenesis.

## 2. Materials and Methods

### 2.1. Microbiological and Molecular Techniques

The bacterial strains used in this study are shown in Table 1 and Table S1. Rhizobia and *Escherichia coli* were grown as described previously [9]. Briefly, rhizobial and *Escherichia coli* strains were cultured using arabinose–gluconate (AG) [14] and Luria–Bertani (LB) media, respectively. Antibiotics were supplemented to the media or agar plates with appropriate concentrations when required for the culture and selection of mutants. For rhizobial strains, polymyxin (Pol) at 50 µg mL^−1^, kanamycin (Km) and streptomycin (Sm) at 200 µg mL^−1^, and spectinomycin (Sp) at 100 µg mL^−1^. For *E. coli* strains, Km, Sm, and Sp at 50 µg mL^−1^.

The primer sets used for rhizobial mutant constructions are detailed in Table S2. Single-crossover homologous recombination was conducted as described previously [15]. For double-crossover homologous recombination [16], the single deletion mutants of the effector genes were constructed by overlap extension PCR using two primer pairs amplifying the upstream and downstream fragments flanking the genes of interest, respectively. The PCR fragments were then cloned into the plasmid pK18mobsac derived from pK18mob [17] and linearized by *Eco*RI and *Hin*dIII restriction enzymes. The plasmid derivatives were transformed into rhizobia via triparental conjugations [18]. Similarly, double gene deletion mutants were constructed using the single deletion mutants as the backgrounds. The mutants were finally verified by antibiotic resistance, PCR, and inoculation assays.

### 2.2. Plant Assays

The seeds of *Vigna* species used in this study are shown in Table S3. Seeds of *Vigna* species were surface sterilized and germinated as described previously [15]. To optimize seed germination, *Vigna* seeds were sanded prior to surface sterilization. One day after transplantation, seedlings were inoculated with *B. elkanii* strains (1 mL of 107 cells mL^−1^ per seedling). The plants were grown in a plant growth cabinet (LPH-410SP; NK Systems, Co. Ltd., Osaka, Japan) at 25 °C and 70% humidity under a day/night regimen of 16/8 h. The plants used for microscopic analysis were grown using seed pouches (Daiki Rika Co. Ltd., Saitama, Japan) supplemented with nitrogen-free Broughton and Dilworth (B&D) solution [20], with two plants per pouch [21]. The symbiotic phenotypes, including nodule number, fresh nodule weight, and whole fresh plant weight, were examined 35 days post-inoculation (dpi). 

### 2.3. GUS Assay and Microscopic Analysis

The GUS-tagged *B. elkanii* strains were constructed by chromosomal integration of the plasmids pCAM120 [22] via conjugations. Single colonies of the constructed mutants were verified by antibiotic resistance, PCR, and GUS assays using X-Glc (Wako Pure Chemical Industries, Osaka, Japan) in dimethylformamide as a substrate. To avoid unexpected effects possibly caused by transposon random insertions, at least two different GUS-tagged mutants were used for inoculation assays. GUS staining and microscopic analysis were performed as described previously [23]. The basal regions of lateral roots were sampled (~1 cm) to observe infection threads (ITs) and nodule organogenesis events [9,21]. Microscopic observations were conducted under a stereoscopic microscope (BX43; Olympus Corporation, Tokyo, Japan). 

### 2.4. Bioinformatics and Statistical Analyses

The amino acid sequences were aligned using MultAlin [24] or Clustal Omega algorithms. Nuclear localization signals (NLSs) of rhizobial NopLs were predicted using cNLS Mapper software [25]. Phosphorylation sites were computationally identified using the NETPHOS 2 program (3.1 Server) [26]. Gene annotation, genome analyses, and *tts* box searches were conducted as previously described by Nguyen et al. [15]. For phylogenetic analyses, NopL, RhcJ, and TtsI homologs were BLASTX searched against rhizobial and pathogenic bacterial genomic databases. For RhcJ and TtsI, at least five to ten homologs from each species, and five to ten different bacterial species from each genus were selected to construct phylogenetic trees. The amino acid sequences were aligned using the MUSCLE algorithm, and phylogenetic trees were constructed using the neighbor-joining method based on the Poisson model in MEGA version 7.0 with 1000 or 10,000 bootstrap replications. For statistical analyses, one-way analysis of variance (ANOVA) followed by *post hoc* tests (Fisher’s or Tukey’s tests at *p* ≤ 0.05) were conducted using Minitab statistical software version 16.0 for multiple test sample comparisons. A two-tailed Student’s *t*-test was also performed by Microsoft Excel for pairwise comparisons. The *p*-values < 0.05 was considered statistically significant. The sample sizes and replications are detailed in figure and table legends.

## 3. Results

### 3.1. T3SS of B. elkanii USDA61 Played Pivotal Roles in Host-Specific Symbioses with Different Vigna Species and V. mungo Cultivars

*Vigna* species, such as *V. mungo*, *V. radiata*, and *V. unguiculata*, were efficiently nodulated by *Bradyrhizobium* spp., including *B. elkanii* [9], *B. japonicum* [27], and *B. yuanmingense* [28]. We previously reported that T3SS of *B. elkanii* USDA61 abolished *V. radiata* nodulation but highly promoted symbiosis with *V. mungo*, suggesting its important roles in controlling host-species-specific symbiotic interactions [9]. To further understand the diverse and distinct symbiotic functions of T3SS in symbiosis among other *Vigna* species, we inoculated several selected *Vigna* species with the wild-type USDA61 and T3SS-deficient mutant BErhcJ. Interestingly, USDA61 T3SS promoted symbioses with *V. unguiculata* and *V. angularis,* but impaired *V. aconitifolia* and *V. trinervia* (Table 1). However, the T3SS-induced symbiotic effects on these *Vigna* species were not as strong as previously observed in *V. radiata* and *V. mungo* [9], since plant weights were not significantly different between USDA61 and BErhcJ strains (Figure S1).

**Figure 1:**
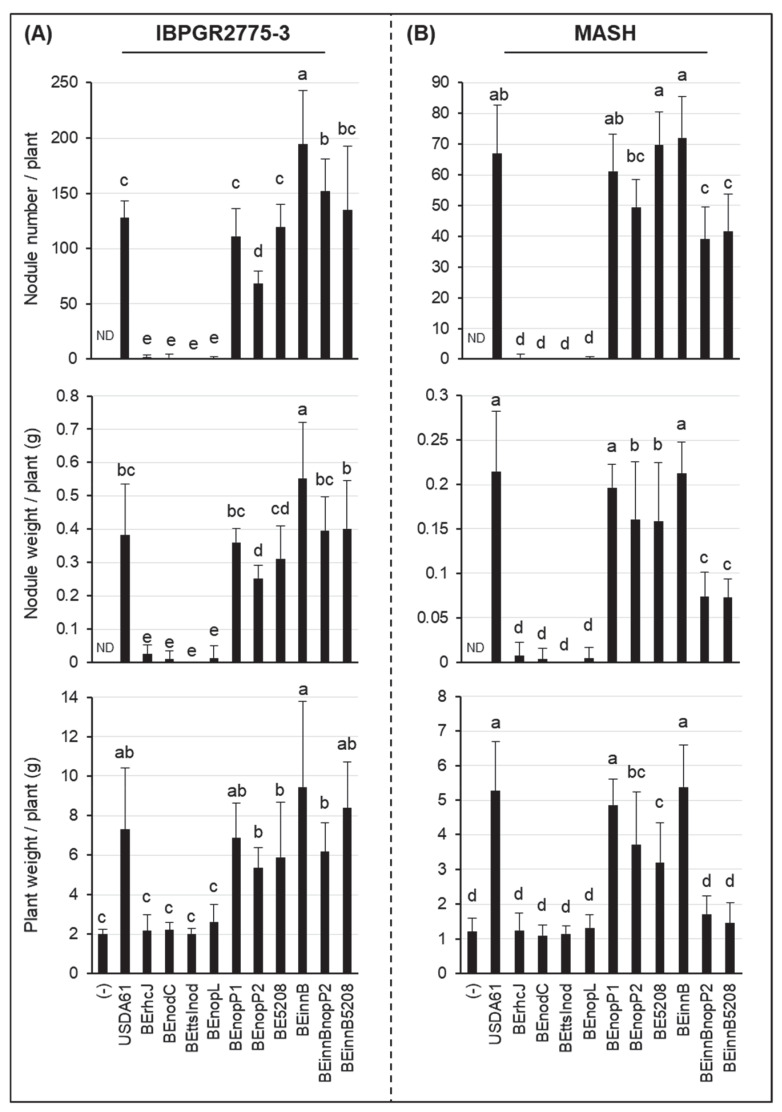
Symbiotic properties of *Vigna mungo* cv. IBPGR2775-3 (**A**) and MASH (**B**) inoculated with *B. elkanii* strains. The data shown are the means of 20 to 30 from five or six independent inoculation assays at 35 dpi. The error bars indicate standard deviations. Means followed by different letters are significantly different at the 5% level (*p* ≤ 0.05 by Tukey’s tests). ND, not detected.

**Figure 2:**
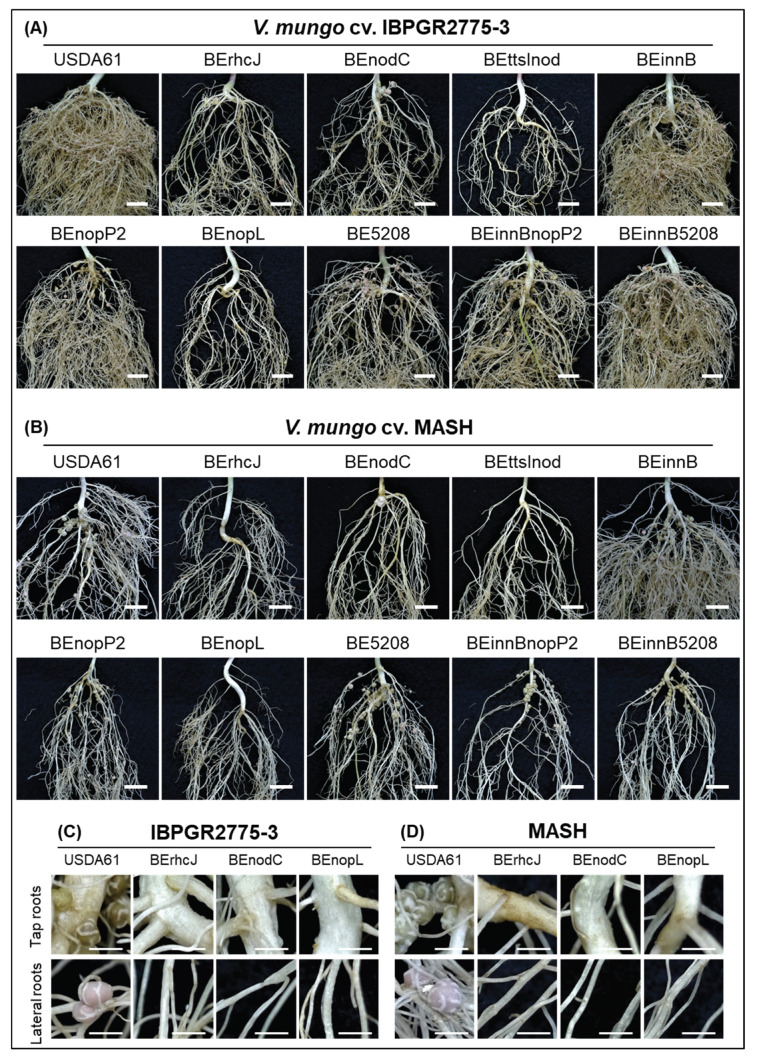
Roots of *V. mungo* cv. IBPGR2775-3 (**A**) and MASH (**B**) inoculated with the *B. elkanii* strains at 35 dpi. Nodulation properties IBPGR2775-3 (**C**) and MASH (**D**). Scale bars: 1 cm for (**A**,**B**) and 0.25 cm for (**C**,**D**).

To further explore the T3SS-induced cultivar-specific nodulation, we conducted inoculation assays using a collection of *V. mungo* cultivars from different countries (Table S3). The results revealed that USDA61 T3SS exhibited compatibility and incompatibility depending on the *V. mungo* cultivars. Indeed, T3SS was responsible for negative effects on the nodulation of *V. mungo* cultivars VM3003, U-THONG2, and CQ5785, but promoted symbiosis with PI173934, MASH, IBPGR2775-3, MAFF2002M3, and CQ5785 (Table 2, Figure S2). Strikingly, among these *V. mungo* plants, inactivation of the T3SS abolished nodulation on IBPGR2775-3 and MASH occurred (Figure 1 and Figure 2). Furthermore, mutations on T3SS or *nodC* exhibited similar nodulation phenotypes, comparable to those of the *ttsI*/*nodC* double mutant BEttsInod, suggesting that T3SS and NFs were both key determinants in host-specific symbiosis with *V. mungo*.

### 3.2. Identification of B. elkanii USDA61 T3Es Involved in Determining Symbiotic Efficiency on V. mungo

To further identify T3Es determining *V. mungo* symbiosis, symbiotic phenotypes of the T3E mutants were investigated using inoculation assays. *B. elkanii* possessed at least two *nopP* gene copies, designated *nopP1* (locus tag *BE61_77110*) and *nopP2* (locus tag *BE61_80730*) (Data S1). NopP1 likely did not function in symbiosis, whereas NopP2 promoted nodulation on IBPGR2775-3 and MASH, as the deletion of *nopP2* significantly reduced nodulation and plant biomass (Figure 1 and Figure 2). Moreover, we identified Bel2-5 as another T3E functioning positively in symbiosis with *V. mungo*. However, its positive effects were likely weak for PI173934 (Figure S3), compared to IBPGR2775-3 (Figure 1A). Noticeably, double deletions of *innB*/*nopP2* or *innB*/*bel2-5* enhanced nodulation of IBPGR2775-3 and significantly reduced MASH nodulation, as compared to the single mutants of *nopP2* and *bel2*-5. Although *nopP2*, *bel2-5,* and *innB* are beneficial for nodulation on PI173934, their double deletion mutants could not restore the T3SS mutant-induced phenotypes (Figure S3).

Although *B. elkanii* USDA61 could efficiently nodulate *V. mungo*, a mixture of pink and white nodules was typically observed on the roots of IBPGR2775-3 at 35 dpi—the tap (white) and lateral (pink) root nodules (Figure 2C). In MASH, nodules were primarily formed around the tap roots, but most exhibited a white color observed at 35 dpi, indicating that early nodulation and nitrogen fixation or weak nitrogenase activities probably occurred (Figure 2D). Such a phenomenon was also observed in the PI173934 roots inoculated with *B. elkanii* strains (Figure S3). Exceptionally, the formation of the lateral root nodules was highly reduced in IBPGR2775-3 plants inoculated with the *nopP2* or *bel2-5* mutant backgrounds (Figure 2A). 

### 3.3. NopL of B. elkanii USDA61 is a Key Determinant for T3SS-Dependent Nodulation in South Asiatic V. mungo Cultivars

Among the T3Es of *B. elkanii* USDA61, NopL served as a key determinant for the T3SS-triggered nodulation of *V. mungo* plants. Impressively, the deletion of *nopL* mimics the T3SS mutant in MASH and IBPGR2775-3 cultivars (Figure 1 and Figure 2). Likewise, the *nopL* and T3SS mutants exhibited comparable symbiotic phenotypes on PI173934 (Figure S3). Noticeably, a few sporadic nodules were formed occasionally on the MASH and IBPGR2775-3 roots inoculated with T3SS, *nopL*, and *nodC* mutants. However, these induced nodules were white or black, indicating inefficient infection. The formation of bump-like structures or immature nodules was also not observed on the roots inoculated with T3SS or *nopL* mutants at 30 dpi (Figure 2).

### 3.4. B. elkanii USDA61 NopL is Required for Early Nodule Development

**Figure 3:**
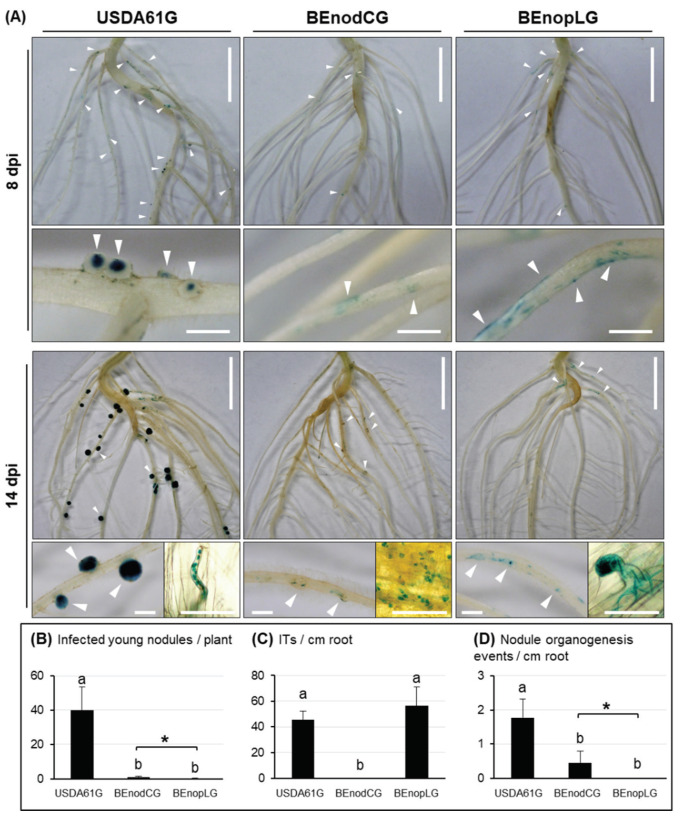
Infection and nodulation properties of *V. mungo* cv. MASH inoculated with the GUS-tagged *B. elkanii* strains. (**A**) Photos of the entire roots, infection threads (ITs), infected nodule primordia, and young nodules at 8 and 14 dpi, respectively. The rhizobial colonization and nodule organogenesis events are marked with white triangles. Scale bars: 1 cm, entire roots; 1 mm, nodule primordia and young nodules; and 50 µm, ITs and rhizobial colonization. Numbers of infected young nodules per plant (**B**), ITs (**C**), and nodule organogenesis events (**D**) induced per cm of the basal regions in lateral roots at 14 dpi. The data shown are means of six or seven plants (three root samples per plant) and the error bars indicate standard deviations. Means followed by different letters are significantly different at the 5% level (*p* ≤ 0.05 by Tukey’s test). “*”, *p* < 0.05 by Student’s *t*-test.

To further explore the NopL functions in the nodulation process, we followed the early infection of *V. mungo* cv. MASH inoculated with GUS-tagged strains of USDA61 (USDA61G), *nodC* mutant (BEnodCG), and *nopL* mutant (BEnopLG) (Figure 3). A number of infected young nodules, infection threads (ITs), and nodule primordia were observed in the roots inoculated with USDA61G. BEnodCG and BEnopLG induced numerous rhizobial colonization sites; however, both failed to induce infected nodule primordia and young nodules as USDA61G. The ITs were not observed in the roots inoculated with BEnodCG; however, BEnopLG could induce IT formation at similar levels as USDA61G. Impressively, several nodule bump-like structures were formed on the roots inoculated with the BEnodCG mutant (Figure 3A), consequently developing young nodules occasionally observed at 14 dpi (Figure 3B). However, such organogenesis events were almost absent in the roots inoculated with the BEnopLG mutant. 

### 3.5. Structural Features of B. elkanii USDA61 NopL

We conducted in silico analyses using several NopL homologs among rhizobia as references. Like other sinorhizobial NopLs, a number of putative phosphorylation sites were also predicted in USDA61 NopL, including seven serine–proline (SP) motifs typical for phosphorylation sites in MAPK (mitogen-activated protein kinase) substrates (Figure S6A) [7]. These phosphorylation sites were primarily distributed in the N-terminal and internal portions, in which the serine (S) residues are dominant (Figure S6A and Data S4). Intriguingly, USDA61 NopL also contained two SP-containing repeats (SPQPDS and SPQPGS) specifically conserved in NopLs of *Sinorhizobium* but not *B. japonicum* and *B. diazoefficiens* (Figure S6A and Data S4). We also identified at least three tandem repeat motifs (3 × SQAGP) in the internal region of USDA61 NopL (Figure S6A and Data S4). Interestingly, alignment analysis verified that these tandem repeat motifs exhibited high similarities to the two catalytic motifs previously identified in *S. fredii* NGR234 NopL [29] (Figure S6B). In addition, the USDA61 NopL, like NGR234 NopL, possessed a nuclear localization signal (NLS) motif situated at the N-terminal Serine-rich region, whereas NopLs of *B. diazoefficiens* USDA110, *B. japonicum* USDA6, and *S. fredii* HH103 did not (Data S3). Notably, NopLs of USDA110 and USDA6 were likely truncated versions lacking the C-terminal portion and functional catalytic domains evolved in the NopLs of USDA61 and NGR234 (Data S4).

### 3.6. Phylogenetic Analyses of Rhizobial NopLs

**Figure 4:**
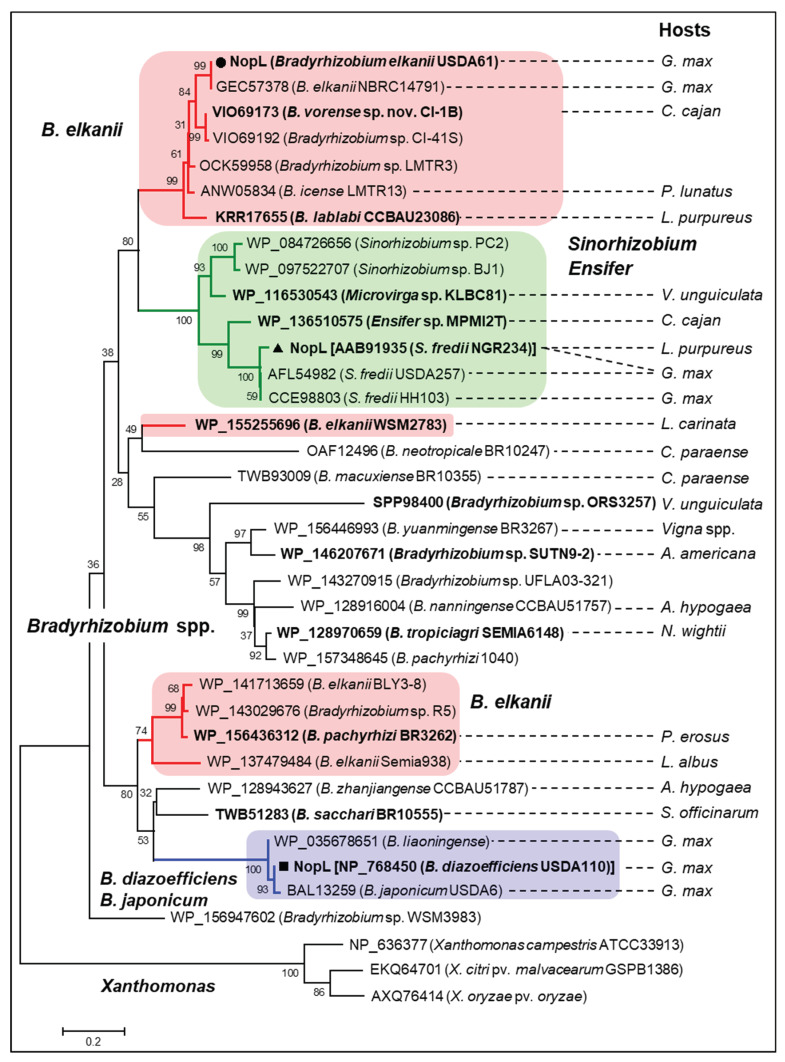
Phylogenetic analysis of *B. elkanii* USDA61 NopL and its homologs among rhizobia. Bootstrap values are expressed as percentages of 10,000 replications. The groups of *B. elkanii* (red), *B. japonicum*/*B. diazoefficiens* (blue), and *Sinorhizobium*/*Ensifer* (green) NopLs are highlighted. The *Xanthomonas* DNA polymerase III subunit γ/τ is used as an outgroup. The main hosts or hosts where rhizobial strains were first isolated are shown: *A. americana*, *Aeschynomene americana*; *A. hypogaea*, *Arachis hypogaea* (peanut); *C. cajan*, *Cajanus cajan* (pigeon pea); *C. paraense*, *Centrolobium paraense*; *G. max*, *Glycine max* (soybean); *L. albus*, *Lupinus albus* (lupin); *L. carinata*, *Leobordea carinata* (leptis); *L. purpureus*, *Lablab purpureus*; *N. wightii*, *Neonotonia wightii* (perennial soybean), *P. erosus*, *Pachyrhizus erosus* (jicama/Mexican yam bean); *P. lunatus*, *Phaseolus lunatus* (lima bean); *S. officinarum, Saccharum officinarum* (sugarcane); and *V. unguiculata*, *Vigna unguiculata* (cowpea).

To further understand the evolutionary history of NopL, phylogenetic analysis using NopL homologs identified among bacterial groups was performed. Of interest, NopL were exclusively conserved rhizobia, dominantly bradyrhizobia, whereas several were identified in *Sinorhizobium*, including *S. fredii*, *Ensifer*, and *Microvirga* (Figure 4). Noticeably, these rhizobia could nodulate efficiently and/or be the main symbionts of *Vigna* plants. No NopL homolog has been identified among *Mesorhizobium* spp., *Rhizobium* spp., or *B. elkanii* strains USDA76 and USDA94, the two closest relatives of USDA61. Surprisingly, the USDA61 NopL group was phylogenetically related to the NopLs of *S. fredii, Ensifer*, and *Microvirga*. In contrast, NopL homologs of the group comprised of *B. diazoefficiens*, *B. japonicum*, and *B. liaoningense* were separated from another *B. elkanii* NopL group excluding the USDA61 NopL. Among the homologs, USDA61 NopL was relatively close to NopLs of *B. elkanii* NBRC14791 (a soybean symbiont) and *B. vorense* sp. nov. CI-1B (a symbiont of *Cajanus cajan*, pigeon pea).

## 4. Discussion 

In this study, we characterized symbiotic interactions between *B. elkanii* and *V. mungo* where both T3SS and NFs play essential roles, and the absence of NFs or T3SS abolished nodulation. Of particular interest, NopL of USDA61 triggered early infection and nodule organogenesis on *V. mungo* in an NF-dependent manner (Table 2). The NFs induced IT formation, whereas NopL ensured efficient early infection and nodule formation in an NF-dependent manner (Figure 3). Notably, nodule primordia-like structures occasionally formed on *V. mungo* roots inoculated with the *nodC* mutant, but not the *nopL* mutant (Figure 3A), suggesting that NopL might also be capable of triggering nodulation in the absence of NFs, although with very low efficiency.

Previous studies have reported that NopL of *S. fredii* NGR234 phosphorylated by host MAPK in vitro [30] interfered with MAPK signaling *in planta* and suppressed premature nodule senescence [29]. In addition, rhizobial NopLs have also exhibited weak similarities to DNA polymerase III subunits γ and τ domain (PRK07764) [7]. However, how NopLs are involved in DNA interactions remain unknown. Our in silico analyses revealed high similarities between NopLs of USDA61 and NGR234, especially in phosphorylation sites/motifs and catalytic domains (Figure S6, Data S4A,B), suggesting their evolutionarily conserved functions. Notably, inactivation of *nopL* excluded nodulation on IBPGR2775-3 and MASH (Figure 1 and Figure 2) but largely reduced nodule numbers of PI173934 (Figure S3). These results suggest that USDA61 NopL, possibly together with other T3Es, suppresses MAPK-triggered responses and/or modulates NF-dependent symbiosis signaling toward determining the nodulation of *V. mungo*, depending on host cultivars (Figure S7). Therefore, NopL might act toward other T3Es or be involved in key steps of the symbiotic process. *B. diazoefficiens* USDA110 and *B. japonicum* USDA6 also recruit at least one NopL copy. However, these NopLs are remarkably different to NopLs of USDA61 and NGR234 in terms of the phylogenetic relationship, length, and evolved functional motifs (Figure 4 and Data S4C), partly reflecting their differentiated functions. 

Inactivation of other T3Es, such as NopP2, Bel2-5, and InnB, also impacts symbiotic effectiveness depending on host cultivar (Figure 1, Figure 2 and Figure S3). Previous studies have revealed phosphorylation activities of the sinorhizobial NopP in vitro [31]. Moreover, at least three NopP2 spots with different pIs were previously detected in the extracellular proteins of USDA61 (Data S2) [11], indicating that NopP2 was possibly phosphorylated in rhizobial cultures post secretion. Hypothetically, NopP2 may be phosphorylated by an as-yet-unknown host kinase to suppress host defense responses and/or promote later nodulation of *V. mungo* (Figure S7). Unlike NopP2, Bel2-5 contains a C-terminal ubiquitin-like protease (Ulp) domain exhibiting similarity to *Xanthomonas* XopDs [10], suggesting that protease activities might be involved in Bel2-5 symbiotic functions. The ability to promote nodulation on *V. mungo* cultivars indicated that Bel2-5 may modulate host symbiosis signaling and/or suppress host defense responses at different levels, depending on host genotypes (Figure S7). Differing from NopP2 and Bel2-5, InnB was a specific “double-edged sword” in symbiosis with *Vigna* species and *V. mungo* cultivars. Certain dual effects of InnB were also highly dependent on host genotypes and correlated with the presence/absence of NopP2 and Bel2-5. In *S. fredii* HH103, NopL and NopP were detrimental to symbiosis with soybean. Conversely, NopL exhibited a negative effect on nodulation on cowpeas, whereas NopP promoted nodulation [32]. However, double deletion of HH103 *nopL* and *nopP* largely caused a decline in nodulation on both soybean and cowpea [32]. Similarly, our results represent the distinct and diverse functions of a T3E cocktail in plant cells (Figure S7); thus, the absence of a T3E may affect T3E-triggered signaling pathways directly or indirectly, resulting in enhanced or reduced nodulation.

Although exhibiting T3SS-determined nodulation, differences in nodulation properties and morphological characteristics of the seed, seedling, and stipule were observed among the three South Asiatic *V. mungo* cultivars (Figure S5). PI173934 and IBPGR2775-3 exhibited some similarities in plant and seed features; however, their stipule morphologies were remarkably different. Interestingly, MASH possessed morphological features of seeds and seedlings that differed from those of PI173934 and IBPGR2775-3. However, the stipule features of MASH were morphologically similar to those of IBPGR2775-3 (Figure S5). These morphological variations may partly reflect the common and distinct traits evolved among these *V. mungo* plants, potentially related to their symbiotic relationships with specific rhizobial strains and T3Es. Our observations of nodulation properties also strengthened the knowledge of the distinct differences among these cultivars (Figure 2 and Figure S3). Genome analyses of these *V. mungo* cultivars will reveal relationships among domestication, morphological evolution, and symbiotic phenotypes of *V. mungo* cultivars.

Phylogenetic analyses revealed that the T3SS of USDA61 is relatively close to those of *B. elkanii*, *B. pachyrhizi*, and *B. jicamae* (Figure S4); the latter two species are novel bradyrhizobia isolated from *Pachyrhizus erosus* (Yam bean/Jicama) [33]. Although co-localized in T3SS, USDA61 NopL was phylogenetically close to its homologs in *S. fredii* (*Ensifer*) (Figure 4), representing their shared functional features and/or similar origins. Surprisingly, NopL homologs have not been identified among genomic databases of *Mesorhizobium* spp., *Rhizobium* spp., or *B. elkanii* strains USDA76 and USDA94, suggesting that NopL is likely not required for the symbiosis of these rhizobia. Our results found that the USDA61-type T3SS was probably horizontally transferred among *B. elkanii*, *B. pachyrhizi*, and *B. jicamae* species as the closest relatives [33]. On the other hand, *nopL* and other T3E genes might be recruited separately during evolution events and symbiosis with different host legumes. 

Rhizobial T3SS (T3Es) and NFs were found to be the key determinants for alternative symbiosis with *V. mungo* plants. For decades, it has been believed that rhizobial NFs play an essential role in activating infection and symbiosis signaling pathways for nodulation on legumes [34]. We previously reported that *B. elkanii* T3SS activated nodulation signaling in soybean [19]. Interestingly, the NF-deficient *B. elkanii* mutant induced bump-like structures that were not evident on plants inoculated with T3SS mutants [19]. The findings in this study advance our understanding that both T3SSs and NFs serve as key determinants for efficient symbiosis with *V. mungo* plants (Figure 2 and Figure 4). Such T3SS-triggered symbiosis has not previously been observed in *G. max* [19], *V. unguiculata* [32], *V. radiata* [11,15], *L. japonicus* [35], or *Aeschynomene* spp. [36]. Furthermore, expressions of the T3SS-related, T3E, and *nod* genes were highly regulated by the common activator NodD and legume flavonoids [18], showing that T3SS (T3Es) and NFs might be simultaneously co-expressed during symbiotic interactions with host legumes. Collectively, these findings suggest that T3SS and NFs might be mutualistically co-evolved in rhizobia, regulated by symbiosis-related common regulators, and act synergistically toward triggering and maintaining efficient nodulation.

## 5. Conclusions

Here, we demonstrated diverse roles of *B. elkanii* T3SS in symbiosis with several *Vigna* species. Of particular interest, we demonstrated that the T3SS of *B. elkanii* USDA61 is essential for symbiosis with at least two cultivars of *V. mungo*. Among the T3Es, NopL served as a key player for inducing nodule primordia. Meanwhile, NF synthesis was required for infection thread formation. These findings provide useful insights into the genetic basis of host specificity in the rhizobium-legume symbiosis.

## Figures and Tables

**Table 1:** *Bradyrhiozbium elkanii* strains used for inoculation tests.

Strains	Characteristics ^a^	References
USDA61	Wild-type strain, Pol^r^	USDA ^b^
BErhcJ	USDA61 derivative harboring insertion in *rhcJ* encoding a membrane protein of the type III secretion apparatus, defective in type III protein secretion, Pol^r^, Km^r^, Tc^r^	[11]
BEnodC	USDA61 derivative harboring insertion in *nodC* gene, Pol^r^, Km^r^, Tc^r^	[19]
BEttsInod	USDA61 derivative harboring insertion in *ttsI* and *nodC* genes, Pol^r^, Km^r^, Sm^r^, Tc^r^	[19]
BEnopL	USDA61 derivative with the *nopL* gene deleted via double-crossover, Pol^r^	This study
BEnopP1	USDA61 derivative harboring insertion of the plasmid pSUPSCAKm::*nopP1* in the *nopP1* gene via single-crossover, Pol^r^, Km^r^	This study
BEnopP2	USDA61 derivative with the *nopP2* gene deleted via double-crossover, Pol^r^	This study
BE5208	USDA61 derivative with the *bel2-5* gene deleted via double-crossover, Pol^r^	This study
BEinnB	USDA61 derivative with the *innB* gene deleted via double-crossover, Pol^r^	This study
BEnopL	USDA61 derivative with the *nopL* gene deleted via double-crossover, Pol^r^	This study
BEnopP1	USDA61 derivative harboring insertion of the plasmid pSUPSCAKm::*nopP1* in the *nopP1* gene via single-crossover, Pol^r^, Km^r^	This study
BEnopP2	USDA61 derivative with the *nopP2* gene deleted via double-crossover, Pol^r^	This study
BEinnBnopP2	USDA61 derivative with both *innB* and *nopP2* deleted via double-crossover, Pol^r^	This study
BEinnB5208	USDA61 derivative with both *innB* and *bel2-5* deleted via double-crossover, Pol^r^	This study

^a^ Pol^r^, polymyxin resistant; Km^r^, kanamycin resistant; Sm^r^, streptomycin resistant; Sp^r^, spectinomycin resistant; Tc^r^, tetracycline resistant; Tp^r^, trimethoprim resistant; Ap^r^, ampicillin resistant. ^b^ United States Department of Agriculture (USDA), Beltsville, MD.

**Table 2:** Symbiotic properties of *V. unguiculata*, *V. trinervia*, *V. angularis*, *V. aconitifolia*, and *V. mungo* varieties inoculated with *B. elkanii* strains ^a^.

Species/Cultivars	Symbiotic Phenotypes Induced by *B. elkanii* Strains ^b^					Origins	Regions
	USDA61	BErhcJ	BEnodC	BE53/BEinnB	BEnopL		
*V. unguiculata*	++	+	N.O.	+++	N.O.	Myanmar	Southeast Asia
*V. trinervia*	–	++	N.O.	–	N.O.	Myanmar	Southeast Asia
*V. angularis*	++	+	N.O.	+++	N.O.	Japan	East Asia
*V. aconitifolia*	–	+	N.O.	+	N.O.	India	South Asia
*V. mungo*							
PI173934	+++	+	N.O.	++	+	India	South Asia
MASH	++	–	–	++	–	Nepal	South Asia
IBPGR2775-3	+++	–	–	++++	–	Pakistan	South Asia
MAFF2002M3	++	+	N.O.	+	N.O.	Myanmar	Southeast Asia
OSUM745	++	+	N.O.	++	N.O.	Philippines	Southeast Asia
VM3003	–	+	N.O.	–	N.O.	Thailand	Southeast Asia
U-THONG2	–	+	N.O.	–	N.O.	Thailand	Southeast Asia
CQ5785	+	++	N.O.	+++	N.O.	Australia	Oceania

^a^ The data shown are summarized from the inoculation assays. ^b^ Symbiotic phenotypes compared in each host: −, inefficient/restricted nodulation; +, efficient nodulation. The number of “+” indicates nodulation efficiency. N.O., not observed.

## Supplementary Materials

The following are available online at https://www.mdpi.com/2073-4425/11/5/474/s1. Table S1: Bacterial strains and plasmids used in this study. Table S2: DNA oligonucleotide primers used in this study. Table S3: Legume seeds used in this study. Figure S1: Symbiotic properties of several *Vigna* species inoculated with *B. elkanii* strains. Figure S2: Symbiotic properties of several *V. mungo* varieties inoculated with the *B. elkanii* strains. Figure S3: Roots (A) and symbiotic properties (B) of *V. mungo* cv. PI173934 inoculated with the *B. elkanii* strains. Figure S4: Phylogenetic analysis of the T3SS structural component protein RhcJ (A) and transcriptional regulator TtsI (B) of *B. elkanii* USDA61. Figure S5: Morphological characteristics of *V. mungo* cv. PI173934, IBPGR2775-3, and MASH. Figure S6: Structural features of *B. elkanii* NopL. Figure S7: Putative model of T3E-triggered symbiotic interactions with *V. mungo*. Data S1: Nucleotide sequences of *nopL*, *nopPs*, *rhcJ*, and *ttsI* in the *B. elkanii* USDA61 genome. Data S2: Alignment of the NopP1, NopP2, and NopP peptide fragments previously detected in the extracellular proteins of *B. elkanii* USDA61. Data S3: Predictions of nuclear localization signals (NLSs) among rhizobial NopLs. Data S4: Alignment analysis of *B. elkanii* USDA61 NopL and its homologs among bradyrhizobia and sinorhizobia (A), sinorhizobia (B), *B. diazoefficiens*/*B. japonicum* (C), and several selected rhizobia (D).
