# Supplementary material for: Identification of Bradyrhizobium elkanii USDA61 Type III Effectors Determining Symbiosis with Vigna mungo
Source: Genes (Basel). 2020 Apr 27;11(5):474. doi: 10.3390/genes11050474 (PMC7291247; doi:10.3390/genes11050474)
Supplement: Supplementary file 1 [file genes-11-00474-s001.zip › Sup dataset_Nguyen et al_Genes 2020/FigS7_Model for T3Es vs V.mungo(ori).pptx]

## Slide 1
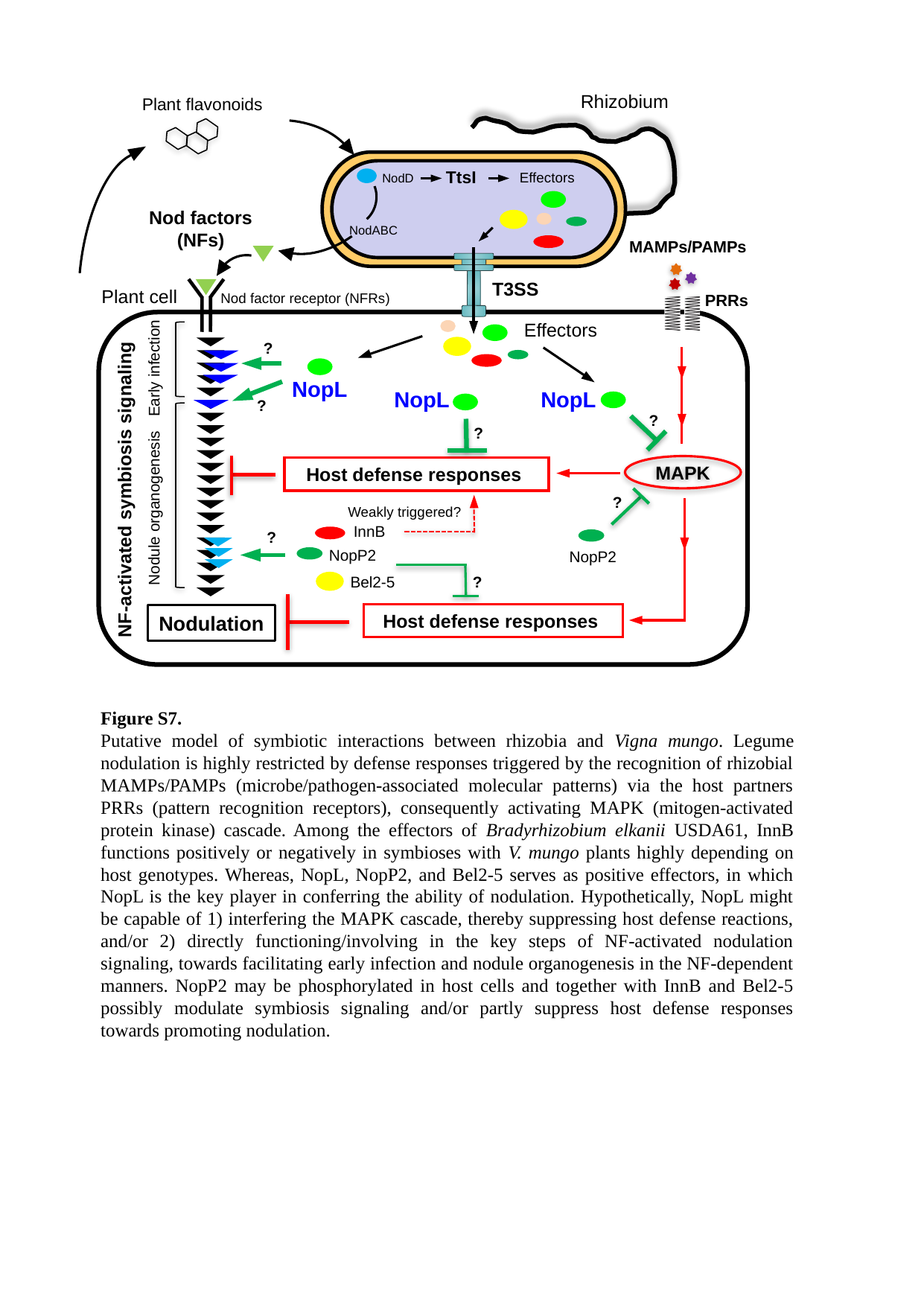

Rhizobium
Plant flavonoids
TtsI
Effectors
NodD
Nod factors (NFs)
NodABC
MAMPs/PAMPs
PRRs
T3SS
Plant cell
Nod factor receptor (NFRs)
Effectors
?
Early infection
NopL
NopL
NopL
?
?
?
MAPK
Host defense responses
NF-activated symbiosis signaling
?
Nodule organogenesis
Weakly triggered?
InnB
?
NopP2
Bel2-5
NopP2
?
Host defense responses
Nodulation
Figure S7.
Putative model of symbiotic interactions between rhizobia and Vigna mungo. Legume nodulation is highly restricted by defense responses triggered by the recognition of rhizobial MAMPs/PAMPs (microbe/pathogen-associated molecular patterns) via the host partners PRRs (pattern recognition receptors), consequently activating MAPK (mitogen-activated protein kinase) cascade. Among the effectors of Bradyrhizobium elkanii USDA61, InnB functions positively or negatively in symbioses with V. mungo plants highly depending on host genotypes. Whereas, NopL, NopP2, and Bel2-5 serves as positive effectors, in which NopL is the key player in conferring the ability of nodulation. Hypothetically, NopL might be capable of 1) interfering the MAPK cascade, thereby suppressing host defense reactions, and/or 2) directly functioning/involving in the key steps of NF-activated nodulation signaling, towards facilitating early infection and nodule organogenesis in the NF-dependent manners. NopP2 may be phosphorylated in host cells and together with InnB and Bel2-5 possibly modulate symbiosis signaling and/or partly suppress host defense responses towards promoting nodulation.
