# Supplementary material for: Identification of Bradyrhizobium elkanii USDA61 Type III Effectors Determining Symbiosis with Vigna mungo
Source: Genes (Basel). 2020 Apr 27;11(5):474. doi: 10.3390/genes11050474 (PMC7291247; doi:10.3390/genes11050474)
Supplement: Supplementary file 1 [file genes-11-00474-s001.zip › Sup dataset_Nguyen et al_Genes 2020/DataS1_Gene sequences.docx]

**Data S1.**

Nucleotide sequences of *nopL*, *nopPs*, *rhcJ*, and *ttsI* in *Bradyrhizobium elkanii* USDA61 genome. The genes (bold) and their promotor sequences are shown. The *nopL* and *nopP2*, but not *nopP1*, is preceded by a conserved *tts* box (bordered).

***nopL* (750 bp)**

CCTGGCATATCAGATCCTGATCGACACCAGGATGAGCCTCGACCGCCTGCTGGAGAGATACTGCAGGGGACGCGCCGCGTCAGGACAGCATCGTGCGCAAGTCTGCGCCAAGCAGAGATGAATTGAAAATGCGAAGCAGCTTGTGGCGAAGGTCAATGCCTTCTCCATTGGCTCATTGCGAATCCTCTTGACGC**TCGTCAGCTTCTCGAAAGCTAACTCTCTTA**GCATGAGAGAGCGGATGTCTAGAGGGCGATGCCGCCTATCCATCTTAATGGAGATGCACATCGAACACCCGATGCTCTGCCGGCGGCTCGGAGGGTGCACTCCACACGAGCCTTCGAAACAGTTCTCTGGAGGTCGCAATGCAA**ATGGCCTTCGTCGAACTATGGCTCGAGCCAAACTTTCAAAGGAGGAGCAGGATGGATTTCAACTCAATCAGCCCAACGAACACAAGCCCGCAGCCCGATTCACCATCAGCGCCCGCGGGTCCAGCGGGCTTTGAGCACCAGCTGCGCGAGGTCGAAGATAGTGCTCTGCCACCTGCTGCGGGATCTCCCGTGCAGCAGGGCAAAGCCTACTCGCCATATCTGGACGCCCGGCATCCCTATTCGCAATATTTGGAGTCGGGGCATCCCTATTCATCACTGTTGGATCGGGAGGATGATCTATATGCGCCGGCTGCGCCCTCCCCCGGGCCATTGGTGGCCGCCAGAGAAAGCTCTCCACAGCCAGGCTCGCAGCAGCCGATCGCTCAAGCCATCGCGGAACTCCCAGAGTTTGATCCTGATCTAATTTGGCAGAATGTGGAAGCCGGGTCATCGCAGGCTGGACCGTCTCAGGCCGGACCTTCGCAAGCCGGGCCATCCTCGTCCGCCGGGGCTGCGCTGTCGGAGCTCACAAATTTCATTCCCGAGGACGAGCGTTTCATAGCCGACCACTGGGTCTTCTGTCCCCATACGGCCTCGGATGCCCAGATCAACATACTAAGGAGGGCCGGCCTCTTGCCTAGCAATAACTCCCGGACCACAAGTTTCACCATGCTGGGTATGCCCCACACAGCCGAATTTCGACAAGAGGGGTTCGTTCGTATCAAACCGTCGATGGACGCGGGCCTCTGA**

***nopP1* (822 bp)**

GCGTTGGTATCCGACAGAACCCGCTTCTTATGACGGCCGATCTAACCGGGGCCAGGCCCCCGATATCCCGTCTCAGATTCTTCCAATTCGTCACGCAGGGACGGGCTGATCTGTAGCGCCTCCGCAATCGAACGAACCGTCTCGCCCGCGTCAGCCCGCGCCATGGCTCATTCGCGGATGTCTTCCGAATGGGGGCGCGTCATCCATGCTGGCCTCCGCCTCCAGCATGGAGGTCGAATCAGAAATCCCACCCGAGGGGAATCCCGATTCCAGAAAACAACAAGCTCCTAGTCAGCTTACGGTCAGTTTCGCCTGGCTACTTTGGCGCGGCCCAAAGCGCCTCAAATCAATAAGAAACCCTCTTGCCTTAGGAGTACGGATCCTC**ATGTACGGTCGAATTGGTGGGTATTATGAAGCAGTTACGTGGGCATCCCACGACGAACACGCGGATGACAGAGATTTCGAGGGCAGGTTCGCTAACATGCACCTGTCCGCAGCGGAGCCGACCTCCTCTTCTGCAGCGCCAACGTATTCCCTCGTGACCAAGCCTCCGATAGAGCCGATCGACAAGGATACGTTTCGCAGAGAAGCAAAGATCTTCCAAAATGATCATGAAATAATGCGCATCGCCGAGAATCCGCGGGAGTACTCGCGTTTCGTATCGACACGAGCCAAGAACGTCAGAGAGGCTGCGGAGGACTACGGCTCGACCAGAGATTCGGAGGAAGCGCGATACTACAGCTATAATCTTGGAAACAAAACTGTCGCACTGCTGCGGACGGAAGGCGGATACAGCATGAACGAATTCCACGACGACAGGTGGCGAGAACTGTTTCCCGGGCGAGAACACATCACCTCCGTCGTCGATCTTCAGCTTGCCCATCCCTTGGTGGAGAACGCAGGCGATATTCTACTCGAATATCAGCTTCGGCGAGATGCGCGAGAAGGCGAGCAACCGTTGCTTAAATGGTATCCACTTAACGAAGAATCGAAAGCTCGCGCGGCGAAGTTAGGTTTCGTAGAGGTCGACGATTGCAATATGGTACTTGATCCTACTCAGCATCCTGACAAATGGACAACGAATAGCGCAGGTGAATGGCAGCGCGCCAACAAACCTGAACGATATCTCGCTAAAGTAGACGACGGCGAGCGTCGTAATACCCATGTAGCAAGCTCCGGATACGCGTACGAGGATGACTTTATGTGA**

***nopP2* (852 bp)**

GCCGAAGGCCGGGTCTGTCCCTACGGCAGGCGTGCGGCATGTAGCGGGCGAGAGCCATTGCTGGTCGCGTGCAACACGGGCGTGAAGATCAGGCCGGCCTGCGCTGATAGTCTCGATGGTCTAGACGACCTGCCGCGGCCCGACGATGCCGCTGCACCGAACGGCGGGGACAGCGCAACAGCGCGTCACGAAATGGTCGATATCGGCAGCACACGACTAAAACGCACAGAACTGGAAAACGAGAGCGTAAGGGCGGGCCGGTCCTCGATAGACGTGTGCATTTGTTCAAGTATCTGATCAAACCCCCAGCCTGCAAGTGCTCTAATGAAAGGCGATCGCGCGTGTGGCGATGAGTGGCCAGTCCTTGAGGGATGGACCATCGTCATATGACGTAATGTGCACTCACCGAATGCAATTCGATGAGTTGCGCGTGCGGCGAATGCGCGCGGAGCGCCTGGTCGTAGCGTGCGAGGCGGACAGAGGCTTTGCGCTATCT**GGGTCAGCTTGCGGTAAGCTAGGCTGTGTA**TTCTAGTCGGCAGAGCTTGCGCGGCACGAGCTCAACAGCGTGGAAATTTGCCGATAGGAGAGCGATCTG**ATGTATGGCAGAATCGTTGGCTCATCGAGCCCGTCCACAGGCGCCAGCCAAGCTGATGAAGCGGGAGAGGCGGGGGACAGCTCGCATTTTACGGAAATGGTTGCAGGCGTCGGTTCAAGTGGGGCGTCGCCGGCGCGATACTCTCTGGAATCCAATCCGCCCATTTCCGAGATCGATCGCTCTTCCTTCAATGACGGACTGTGGAGATTTCTGGGTTCTGATATCCAGAGCATCGCAAGTAGCCGGGAAGAGTACTCGGATTTCGTATCCAAGAAAGCTGAGCGCGCAGCAACGGTCGCTGGAAGCTATCTCCACACCTACGATGATCTGTCCAGGCCAGCGAAATTTTTCAGCTATAAATTGGGTGACGAAACGGTCGGCCTCTTAAGAGTGGGAGGTCCGGTTCGGATCAAAGGAGACGCCTTCCGGAACCAGTTTGGCCGCAACGATCTGACGTCTGTGGTAGACCTTCGGGTGACACATCCCCTGGTCGAGAATGCGGGCGATATTCTGCTGGAACATCAACTGAGAATTGATGCGCGCAACGGCGCTGAGCCGCTGATCTTGTCAAAACCAGCCTTAGGCGGGATGGAACCCCGCCTGGCGGAAATGGGTTTTGTTCACGTGGGGCGAAACCACTGGGTGCTTGATCCTCACCAGCATCCGGAAGTGTGGACCAAGAATGAGAACGATGAGTGGCAGCGAGTAGGCAAGCCTACAAAGTACCTCGCCAAGGCAGGGGATGGTGATAGCGCGACCCAAGCGCCTCGCCAGTTTGACTCTTCTGACGAAGATGATTCGACAGAATACTACTACTTGGAGCGCGCCCTTGCGGGGCTGCACACAGAGTAG**

***rhcJ* (876 bp)**

**ATG**AAGCTGATGCGTGGTGTGATGTGCAGTGCGGGCAACGGCAGCCGTCAATCGTGGCGACGGCTTCGCGTTTGCCTTGCTCTGCCCCTTCTTGTTCCGTTGCTCGGCTGCAAAGCTGATCTCTACAGTAAGATTCAGGAGCGTGAAGCCAATGAGATGCTTGCGCTTCTCCTTGGCAAGGGTGTCGATGCAGCTCGTGTTGTCGCCAAAGATGGGACCAGCACGATCCAGGTCGAGGAAAGGCAGCTCGCCTATTCGATTGACTTGCTGAATGTTGAGGGGCTGCCGCGCCAATCTTTCAAGAATCTTGGCGAGGTATTCAAGGGATCGGGCCTCGTTGCGTCGCCGATCGAGGAGCGGGCCCGTTACGTTTATGCCCTCAGCGAGGAATTGTCGCGCACCATTAGCGATATCGATGGCGTCCTTTCCGCCCGGGTCCATGTGGTCCTTCCTAAAAACGATCTGTTGCGGCAAGATGCGACCCCGTCCTCAGCGTCGGTTTTCATCCGACATGGCTCCAACGCAAAGCTCTCGGCGCTGTTGCCTCAGATCAAGATGCTCGTAGCCAACAGCATCGAAGGGCTGTCCTACGACAAGGTGGCTGTGGTCTTCGTGCCGGTTGAGCGAACTCCGCTTGAGCAGCCAGCGTCGCCGACAGCCGCTTCAGCTCAAAGCGCAAAATCTGCTTCAACGCCGTGGCTTGCGCTTGGGGTTGGAGGCGCCGGCGCCATATTCGTCATTGCATCTTATGTGTTGCTCGGTGCGCGTCTTCGTCAGTTCGGGCAATCATCGCGCAACCTGATCATGTTCAACAGGCGTTCGAATGTGCCCGCCGTTCAGGCTGCTGGTAAAAAGATCATGTCTGATTCGACA**TAG**

***ttsI* (690 bp)**

**ATG**CGAATTCTCCTGGTTGATCATCATGCGGACTTTGCCCGTGCTGTGAAGGAAGCGCTCCCCTATTGCGGGTTCGCAGTTGACGTGACACGCACGCTGGATGAGGCGGCGGCCGCGCTGGATTGCGCCAACTATCACATTCTTTTGCTCGAATTGGTTCTGCCCGATGGAGACGGCTTGGATTGGCTGAAGCAGCTGCGGCGCGAGGGACGCTCGATGCCGGCCATTATGATGAGCAGTCTCAACGATCTCGGCCGGCGGATTGCGATCTTCAATGCGGGCGCGGACGATTTCCTCCCCAAACCCGTATCGACCGAGGAACTCATCGCACGCATGCGGGCCATTCTGCGGCGGTCGACGCAAATGACGGCGCCGCTCGTGACATTCGGCAATCTGCACTTCGACCCCATTGCGAGGCAAGTCGCGGTCGGTGGTCGGATACTGAAGATTGCCCGCCGCGAAGTGTGCATTCTTGAACATTTGCTCAACCGTGCCGGCCGCACCGTGCCGCGCGCATCACTGGAGGACAGCCTGTACGCGTTCGACGATGAGGTCTCGACCAATGCGCTGGAAGTCGGGATCTATCGCCTGCGCACGCATTTGAGCCAGTCGGGTGCGACGCTCAGGATCAAGACCGCGCGCGGCGTCGGTTACACCCTTGAACTCATTGAGGCAGCCTCGGCTGCC**TGA**
