# Supplementary material for: Identification of Bradyrhizobium elkanii USDA61 Type III Effectors Determining Symbiosis with Vigna mungo
Source: Genes (Basel). 2020 Apr 27;11(5):474. doi: 10.3390/genes11050474 (PMC7291247; doi:10.3390/genes11050474)
Supplement: Supplementary file 1 [file genes-11-00474-s001.zip › Sup dataset_Nguyen et al_Genes 2020/FigS6_Genome context&NopL features(ori)_2.pptx]

## Slide 1
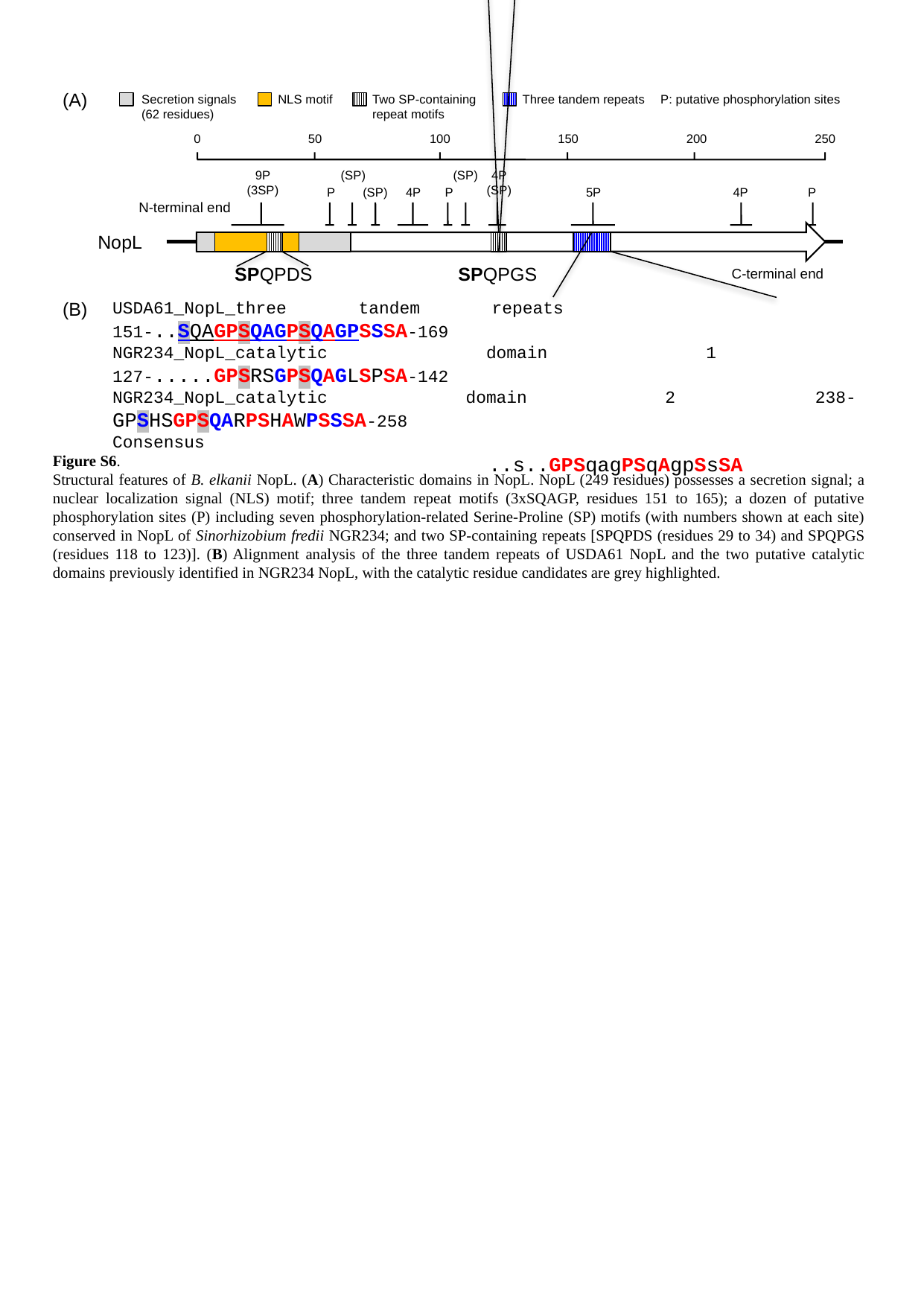

(A)
Secretion signals
(62 residues)
NLS motif
Three tandem repeats
P: putative phosphorylation sites
Two SP-containing repeat motifs
0
50
100
150
200
250
9P
(3SP)
(SP)
(SP)
4P
(SP)
P
(SP)
4P
P
5P
4P
P
N-terminal end
NopL
SPQPDS
SPQPGS
C-terminal end
USDA61_NopL_three tandem repeats 		151-..SQAGPSQAGPSQAGPSSSA-169
NGR234_NopL_catalytic domain 1		127-.....GPSRSGPSQAGLSPSA-142
NGR234_NopL_catalytic domain 2		238-GPSHSGPSQARPSHAWPSSSA-258
Consensus								 	 ..s..GPSqagPSqAgpSsSA
(B)
Figure S6.
Structural features of B. elkanii NopL. (A) Characteristic domains in NopL. NopL (249 residues) possesses a secretion signal; a nuclear localization signal (NLS) motif; three tandem repeat motifs (3xSQAGP, residues 151 to 165); a dozen of putative phosphorylation sites (P) including seven phosphorylation-related Serine-Proline (SP) motifs (with numbers shown at each site) conserved in NopL of Sinorhizobium fredii NGR234; and two SP-containing repeats [SPQPDS (residues 29 to 34) and SPQPGS (residues 118 to 123)]. (B) Alignment analysis of the three tandem repeats of USDA61 NopL and the two putative catalytic domains previously identified in NGR234 NopL, with the catalytic residue candidates are grey highlighted.
