# Supplementary material for: Identification of Bradyrhizobium elkanii USDA61 Type III Effectors Determining Symbiosis with Vigna mungo
Source: Genes (Basel). 2020 Apr 27;11(5):474. doi: 10.3390/genes11050474 (PMC7291247; doi:10.3390/genes11050474)
Supplement: Supplementary file 1 [file genes-11-00474-s001.zip › Sup dataset_Nguyen et al_Genes 2020/DataS4_Align NopLs vs rhizobial homologs.docx]

**Data S4.**

Alignment analysis of *Bradyrhizobium elkanii* USDA61 NopL and its homologs among bradyrhizobia and sinorhizobia (**A**); sinorhizobia (**B**); *B. diazoefficiens*/*B. japonicum* (**C**); and several selected rhizobia (**D**). The alignment was done using Clustal Omega algorithm. The “*” indicates perfect alignment, the “:” and “.” indicate sites belonging to the group exhibiting strong and weak similarity, respectively. In (**A**), the 62 N-terminal amino acid residues are shown (bordered). In (**A**–**C**), nuclear localization signal (NLS) motifs (grey) are highlighted. No NLS motif is predicted in NopLs of *B. diazoefficiens* USDA110, *B. japonicum* USDA6, and *Sinorhizobium fredii* HH103. The computationally predicted phosphorylation residues (Serine, S; Threonine, T; and Tyrosine, Y) in NopLs of USDA61 (249 residues), USDA110 (167 residues), and *S. fredii* NGR234 (338 residues) are blue. In (**B**), the four phosphorylated residues (S89, S139, S148, and S198) biochemically verified in NGR234 NopL are yellow highlighted; and the other S residues essential for NGR234 NopL functions [1] are blue highlighted. The seven Serine-Proline (SP) of USDA61 NopL are double underlined. The two SP-containing repeats and three tandem repeat motifs (3xSQAGP) of USDA61 NopL are underlined by blue and green, respectively. The two tandem repeats previously identified in NGR234 NopL is underlined with black and red, respectively [2]. The three putative catalytic domains of NGR234 NopL [2] are orange highlighted in (**A**) and (**B**), with the catalytic residue candidates are bordered.

(**A**) **USDA61 NopL, bradyrhizobial and sinorhizobial NopLs**

**NopL_BeUSDA61** MAFVELWLEPNFQRR**S**RMDFN**S**I**S**P**T**N**TSPQPDS**PSAPAGPAGFEHQLREVED**S**ALPPAA 60

**NopL_BdUSDA110** -----------------MDFNAVAPAN**TSPEPDT**AR**T**A**T**DA**T**EFERQL**S**G**S**EAPASAQGV 43

BAL13259_BjUSDA6 -----------------MDFNAVAPANT**SPEPD**TARTATDATEFERQLSGSEAPASAQGV 43

**NopL_SfNGR234** -----------------MDIN**S**T**S**PLNA**SPQPDS**PPP-ANASAFAHQLSGFQY**S**-PPHAA 41

CCE98803_SfHH103 -----------------MDINSTRPLNA**SPQPD**SPPP-ANESAFAHQLSGFQYS-PPHAA 41

**:*: * *:**:**: :. : * :** : ..

**NopL_BeUSDA61** G**S**PVQQGKAYSPYLDARHPY**S**QYLE**S**GHP**Y**S**S**LLDREDDL**Y**------------------- 101

**NopL_BdUSDA110** AHPVLQGEAYSP**Y**LDAGHPY**S**PYLE**T**GHLYPPYPDLAHPLGPD----------------- 86

BAL13259_BjUSDA6 AHPVLQGEAYSPYLDAGHPYSPYLETGHLYPPYPDLAHPLGPD----------------- 86

**NopL_SfNGR234** DSLLPQVEAD**S**PY**L**DTRHPY**S**Q**Y**LD**S**AYPYP**S**PCEWQHDL**YT**RTRER**S**PHP**S**EQRPHARV 101

CCE98803_SfHH103 DSLLPQVEADSPYLDTGHPYSQYLDSAYPYPSPCEWQHDLYTRTRERSPHPSEQRPHARV 101

: * :* *****: **** **::.: * : . *

**NopL_BeUSDA61** ------------------------------------------------------------ 101

**NopL_BdUSDA110** ------------------------------------------------------------ 86

BAL13259_BjUSDA6 ------------------------------------------------------------ 86

**NopL_SfNGR234** LQGAPEHDQDQHLEAAGPREG**S**WQVGP**S**R**S**GP**S**QAGL**S**P**S**ATPLNP**S**PPPHA**T**DLE**T**KHP 161

CCE98803_SfHH103 LQDAPEHDQDQHVEAAGPRAGSWQVGPSRSGPSQAGPSSSATPLNASPPPHATDLETEHP 161

**NopL_BeUSDA61** --------------------APAAP**S**PGPLVAARE**SSPQPGS**QQPIAQAIAELPEFDPDL 141

**NopL_BdUSDA110** ------------SGWQDNL**Y**AAPAAVAAP---EPDNGQ**Q**HL**S**PQAIAQAIEEHPGFDQDV 131

BAL13259_BjUSDA6 ------------SGWQDNLYAAPAAVAAP---EPDNGQLHL**S**PQAIAQAIEEHPGFDQDV 131

**NopL_SfNGR234** Y**S**QYLDWANPSLLDWQQDLH**T**RA**T**A**S**PAPLTAERGR**SPQPS**EQQPHARA-LQVPEYDQDL 220

CCE98803_SfHH103 YSQYLDWANPSLLDWQHDLHTRATASPAPLTAERGK**SPQP**SEQQPHARA-LQVPEYDQDL 220

: : .* . . * *:* : * :* *:

**NopL_BeUSDA61** IWQNVEAGS**SQAGP----SQAGPSQAG----------PSS**SAGAALSELTNFIPEDERFI 187

**NopL_BdUSDA110** IWQ**T**LDVGP**S**E**A**E**P**RHGEP**Q**A**GTS**HA**G**P**S**RTAPF**S**G------------------------ 167

BAL13259_BjUSDA6 IWQTLDVGPSE**A**E**P**RHGEP**Q**A**G**T**S**HA**G**PSRTAPFSG------------------------ 167

**NopL_SfNGR234** IWQRVDAAGPQ**A**G**P**----W**Q**V**G**P**S**HS**G**P**S**QARP**S**HAWPS**S**SAGAEPAEL**S**DFVM**D**SGVRA 276

CCE98803_SfHH103 IWQRVDAAGPQ**A**G**P**----W**Q**V**G**P**S**HS**G**PSQARPSHAWPSSSAGAEPTELSDFVMDSGVRA 276

*** ::.. :* * *.* *::*

**NopL_BeUSDA61** ADHWVFCPHTASDAQINILRRAGLLP**S**NN**S**RT**TS**FTMLGMPHTAEFRQEGFVRIKP**S**MDA 247

**NopL_BdUSDA110** ------------------------------------------------------------ 167

BAL13259_BjUSDA6 ------------------------------------------------------------ 167

**NopL_SfNGR234** WDHWFLAPHMASEDQM**S**MLRA**T**GLMPTAEVPTTTFLMMGMRHVAEFRGEGVIRIRP**S**VDF 336

CCE98803_SfHH103 WDHWFLAPHMASEDQMSMLRATGLMPTAEVPTTTFLMMGMPHVAEFRGEGVIRIRPSLDF 336

**NopL_BeUSDA61** GL 249

**NopL_BdUSDA110** -- 167

BAL13259_BjUSDA6 -- 167

**NopL_SfNGR234** DI 338

CCE98803_SfHH103 DI 338

(**B**) **USDA61 NopL and sinorhizobial NopLs**

**NopL_BeUSDA61** MAFVELWLEPNFQRR**S**RMDFN**S**I**S**P**T**N**TSPQPDS**PSAPAGPAGFEHQLREVED**S**ALPPAA 60

**NopL_SfNGR234** -----------------MDIN**S**T**S**PLNA**SPQPDS**PPP-ANASAFAHQLSGFQY**S**-PPHAA 41

CCE98803_SfHH103 -----------------MDINSTRPLNA**SPQPDS**PPP-ANESAFAHQLSGFQYS-PPHAA 41

**:** * *:******* *. :.* *** .: * * **

**NopL_BeUSDA61** G**S**PVQQGKAYSPYLDARHPY**S**QYLE**S**GHP**Y**S**S**LLDREDDL**Y**------------------- 101

**NopL_SfNGR234** DSLLPQVEAD**S**P**Y**LDTRHPY**S**Q**Y**LD**S**AYPYP**S**PCEWQHDL**YT**RTRER**S**PHP**S**EQRPHARV 101

CCE98803_SfHH103 DSLLPQVEADSPYLDTGHPYSQYLDSAYPYPSPCEWQHDLYTRTRERSPHPSEQRPHARV 101

.* : * :* *****: *******:*.:** * : :.***

**NopL_BeUSDA61** ------------------------------------------------------------ 101

**NopL_SfNGR234** LQGAPEHDQDQHLEAAGPREG**S**WQVGP**S**R**S**GP**S**QAGL**S**P**S**A**T**PLNP**S**PPPHA**T**DLE**T**KHP 161

CCE98803_SfHH103 LQDAPEHDQDQHVEAAGPRAGSWQVGPSRSGPSQAGPSSSATPLNASPPPHATDLETEHP 161

**NopL_BeUSDA61** --------------------APAAP**S**PGPLVAARE**SSPQPGS**QQPIAQAIAELPEFDPDL 141

**NopL_SfNGR234** Y**S**QYLDWANPSLLDWQQDLH**T**RA**T**A**S**PAPLTAERGR**SPQPS**EQQPHARA-LQVPEYDQDL 220

CCE98803_SfHH103 YSQYLDWANPSLLDWQHDLHTRATASPAPLTAERGK**SPQP**SEQQPHARA-LQVPEYDQDL 220

: *: **.**.* * ****..*** *:* ::**:* **

**NopL_BeUSDA61** IWQNVEAGS**SQAGPSQAGPSQAG----------PSS**SAGAALSELTNFIPEDERFIADHW 191

**NopL_SfNGR234** IWQRVDAAGP**QAGP**W**Q**V**GPS**HS**G**P**S**QARP**S**HAW**P**S**S**SAGAEPAEL**S**DFVM**D**SGVRAWDHW 280

CCE98803_SfHH103 IWQRVDAAGP**QAGP**W**Q**V**GPS**HS**G**PSQARPSHAW**P**SSSAGAEPTELSDFVMDSGVRAWDHW 280

***.*:*.. **** *.***::* ******* :**::*: :. ***

**NopL_BeUSDA61** VFCPHTASDAQINILRRAGLLP**S**NN**S**RT**TS**FTMLGMPHTAEFRQEGFVRIKP**S**MDAGL 249

**NopL_SfNGR234** FLAPHMASEDQM**S**MLRA**T**GLMPTAEVPTTTFLMMGMRHVAEFRGEGVIRIRP**S**VDFDI 338

CCE98803_SfHH103 FLAPHMASEDQMSMLRATGLMPTAEVPTTTFLMMGMPHVAEFRGEGVIRIRPSLDFDI 338

.:.** **: *:.:** :**:*: : **:* *:** *.**** **.:**:**:* .:

(**C**) **USDA61 NopL and bradyrhizobial NopLs**

**NopL_BeUSDA61** MAFVELWLEPNFQRR**S**RMDFN**S**I**S**P**T**N**TSPQPDS**PSAPAGPAGFEHQLREVED**S**ALPPAA 60

**NopL_BdUSDA110** -----------------MDFNAVAPAN**TSP**E**PDT**AR**T**A**T**DA**T**EFERQL**S**G**S**EAPASAQGV 43

BAL13259_BjUSDA6 -----------------MDFNAVAPANT**SP**E**PD**TARTATDATEFERQLSGSEAPASAQGV 43

****:::*:****:**: : :. : **:** * * ..

**NopL_BeUSDA61** G**S**PVQQGKAYSPYLDARHPY**S**QYLE**S**GHP**Y**S**S**LLDRE----------DDL**Y**APAAP**S**PGP 110

**NopL_BdUSDA110** AHPVLQGEAYSP**Y**LDAGHPY**S**PYLE**T**GHLYPPYPDLAHPLGPDSGWQDNL**Y**AAPAAVAAP 103

BAL13259_BjUSDA6 AHPVLQGEAYSPYLDAGHPYSPYLETGHLYPPYPDLAHPLGPDSGWQDNLYAAPAAVAAP 103

. ** **:******** **** ***:** * * *:*** * .*

**NopL_BeUSDA61** LVAARE**SSPQPGS**QQPIAQAIAELPEFDPDLIWQNVEAGS**SQAGPSQ----AGPSQAGPS** 166

**NopL_BdUSDA110** ---EPDNGQ**Q**HL**S**PQAIAQAIEEHPGFDQDVIWQ**T**LDVGP**S**E**A**E**P**RHGEPQ**AGTS**H**AGPS** 160

BAL13259_BjUSDA6 ---EPDNGQLHL**S**PQAIAQAIEEHPGFDQDVIWQTLDVGP**S**E**A**E**P**RHGEPQ**AG**T**S**H**AGP**S 160

:.. * * ***** * * ** *:***.::.* *:* * : ** *:****

**NopL_BeUSDA61** **S**SAGAALSELTNFIPEDERFIADHWVFCPHTASDAQINILRRAGLLP**S**NN**S**RT**TS**FTMLG 226

**NopL_BdUSDA110** RTAPF**S**G----------------------------------------------------- 167

BAL13259_BjUSDA6 RTAPFSG----------------------------------------------------- 167

:* :

**NopL_BeUSDA61** MPHTAEFRQEGFVRIKP**S**MDAGL 249

**NopL_BdUSDA110** ----------------------- 167

BAL13259_BjUSDA6 ----------------------- 167

(**D**) **USDA61 NopL and several selected rhizobial NopLs**

**NopL_BeUSDA61** MAFVELWLEPNFQRRSRMDFNSISPTNT**SPQPDS**PSAPAGPAGFEHQLREVEDSALPPAA 60

GEC57378_BeNBRC14791 -----------------MDFNSISPTNT**SPQPDS**PSAPAGPAGFEHQLREVEDSALPPAA 43

WP_141713659_BeBLY3-8 -----------------MDFNAIGPANTR**P**E**PD**LPEPSMDATGFEQRLSGAQAGALPQHA 43

WP_137479484_BeSemia938 -----------------MDFNAIGSANAG**P**E**P**EPPEPFTDAPSFEQRLSGAHASAPPQHA 43

WP_155255696_BeWSM2783 ------------------------------------------------------------ 0

NopL_SfNGR234 -----------------MDINSTSPLNA**SPQPDS**PP-PANASAFAHQLSGFQYS-PPHAA 41

CCE98803_SfHH103 -----------------MDINSTRPLNA**SPQPDS**PP-PANESAFAHQLSGFQYS-PPHAA 41

WP_136510575_EspMPMI2T -----------------MDINSTSPLNA**SPQPDS**PP-PANESAFAHQLSGFQYS-PPHAA 41

WP_146207671_BspSUTN9-2 ------------------------------------------------------------ 0

SPP98400_BspORS3257 ------------------------------------------------------------ 0

**NopL_BeUSDA61** GSPVQQGKAYSPYLDARHPYSQYLESGHPYSSLLDRED---------------------- 98

GEC57378_BeNBRC14791 GSPVQQGKAYSPYLDARHPYSQYLESGHPYSSLLDRED---------------------- 81

WP_141713659_BeBLY3-8 APPVLQGGAYSPYLDAGHPYSPYLDAGHPYSPYLDLAHPWP------------------- 84

WP_137479484_BeSemia938 APPVLQGDAYSPYLGAGHPYSPYLDAGHPYSPYLDLAHPST------------------- 84

WP_155255696_BeWSM2783 ---MPQGEAYSPYLEAGHPYSS-------------------------------------- 19

NopL_SfNGR234 DSLLPQVEADSPYLDTRHPYSQYLDSAYPYPSPCEWQHDLYTRTRERSPHPSEQRPHARV 101

CCE98803_SfHH103 DSLLPQVEADSPYLDTGHPYSQYLDSAYPYPSPCEWQHDLYTRTRERSPHPSEQRPHARV 101

WP_136510575_EspMPMI2T DSLLPQVEAYSPYLDTGHPYSQYLDSANPYSSSFEWQDDPYTRARERSPHPSEQQPHARV 101

WP_146207671_BspSUTN9-2 ------------------------------------------------------------ 0

SPP98400_BspORS3257 ------------------------------------------------------------ 0

**NopL_BeUSDA61** ------------------------------------------------------------ 98

GEC57378_BeNBRC14791 ------------------------------------------------------------ 81

WP_141713659_BeBLY3-8 ------------------------------------------------------------ 84

WP_137479484_BeSemia938 ------------------------------------------------------------ 84

WP_155255696_BeWSM2783 ------------------------------------------------------------ 19

NopL_SfNGR234 LQGAPEHDQDQHLEAAGPREGSWQVGPSRSGPSQAGLSPSATPLN--------------- 146

CCE98803_SfHH103 LQDAPEHDQDQHVEAAGPRAGSWQVGPSRSGPSQAGPSSSATPLN--------------- 146

WP_136510575_EspMPMI2T LQQVPEPDQDQHVEAAGTQAGSWQVGPSRSGPSQAGPSSSANPLNERPQPADERPFAQQL 161

WP_146207671_BspSUTN9-2 ------------------------------------------------------------ 0

SPP98400_BspORS3257 ------------------------------------------------------------ 0

**NopL_BeUSDA61** ------------------------------------DLYAPAAPSPGPLVAARES**SPQPG** 122

GEC57378_BeNBRC14791 ------------------------------------DLYAPAAPSPGPLVAARES**SPQP**G 105

WP_141713659_BeBLY3-8 ------------------------------DLDRENDPFTP---SAEHVAPEPES-**PQ**YL 110

WP_137479484_BeSemia938 ------------------------------DLGWEDNSHTP---TADHIAPELESF**PQP**L 111

WP_155255696_BeWSM2783 ------------------------------HLNWQDDLHTPAAVSPEPLPATGESFQ**QP**S 49

NopL_SfNGR234 ----PSPPPHATDLETKHPYSQYLDWANPSLLDWQQDLHTRATASPAPLTAERGR**SPQP**S 202

CCE98803_SfHH103 ----ASPPPHATDLETEHPYSQYLDWANPSLLDWQHDLHTRATASPAPLTAERGK**SPQP**S 202

WP_136510575_EspMPMI2T SGFQDSPPPHATDLGTEHPYSQYLDWANPSSLDWRHDLYTRATASPSPLAAERER**SPQP**G 221

WP_146207671_BspSUTN9-2 -------------------------------MDF--NALSRADNSPASHP--SSSPAG**P**R 25

SPP98400_BspORS3257 ------------------------------------------------------------ 0

**NopL_BeUSDA61** **S**QQPIAQAIAELPEFDPDLIWQNVEAGS**SQAG--PSQAG----------P**---------- 160

GEC57378_BeNBRC14791 SQQPIAQAIAELPEFDPDLIWQNVEAGS**S**QAG--P**S**QAG----------P---------- 143

WP_141713659_BeBLY3-8 PQQTIAQAIEAHPGVDQDLIWQNVAG-P**S**HAG--P**S**YAGPLLEGPSHAGP---------- 157

WP_137479484_BeSemia938 SQQTIAQAIEAHPDIDQDLIWQNVDAGP**S**GAGPSP**S**HAGPSYAGLVQARP---------- 161

WP_155255696_BeWSM2783 SQQAIAEPLAKAAYFDQDLIWQELESG-LQAG--P**S**QAGPSSFALEGPQRPISQQPLAQS 106

NopL_SfNGR234 EQQPHARAL-QVPEYDQDLIWQRVDAAGPQAG--PWQVG----------P---------- 239

CCE98803_SfHH103 EQQPHARAL-QVPEYDQDLIWQRVDAAGPQAG--PWQVG----------P---------- 239

WP_136510575_EspMPMI2T EQQPHARAL-QVPEHDQDLIWQHVDAAGPQAG--P**S**HAG----------R---------- 258

WP_146207671_BspSUTN9-2 EFERELMELHEIPEYSQDLIWQELGGRS**S**RVEPSL**S**G----------GGRSNSQQPIAQH 75

SPP98400_BspORS3257 --------MHKIPEYSQDLIWQELGAGS**S**RVEPSL**S**G----------GERTNSQQPIAQH 42

: . *****.: . .

**NopL_BeUSDA61** --------------**SQAG-----------PS**S--SAGAALSELTNFIPEDERFIAD---- 189

GEC57378_BeNBRC14791 --------------**S**QAG-----------**PS**S--SAGAALSELTNFIPEDERFIAD---- 172

WP_141713659_BeBLY3-8 --------------**S**HAGP-THARPLQAG**PS**QAGPSETAPPELSEFRMLNGRLAKD---- 198

WP_137479484_BeSemia938 --------------**S**HAGP-SHARPLQAG**PS**EAGPSEATPPELSGFRMVEGRLAKD---- 202

WP_155255696_BeWSM2783 LAEAPDFDKDPVWQELDPG-LQAGPSQAG**PS**S--SAGAAPRELGDFVMNNGRRASE---- 159

NopL_SfNGR234 --------------**S**HSGP-SQARPSHAW**PS**S--SAGAEPAELSDFVMDSGVRAWD---- 278

CCE98803_SfHH103 --------------**S**HSGP-SQARPSHAW**PS**S--SAGAEPTELSDFVMDSGVRAWD---- 278

WP_136510575_EspMPMI2T --------------**S**EAG-----------**PS**S--SAGAEPAELNDFVMDSGVRAWD---- 287

WP_146207671_BspSUTN9-2 RADASDFGNDLVWQDLEPWLMWAGSLHAG**PS**MLHAGVA-SSEIGDFAMANGRRAKD---- 130

SPP98400_BspORS3257 RVDASEFGNDLVWQDPG-------HLNAG**PS**QADSRARTGCSAGTWRFRHGERPSRQGRV 95

. ** . :

**NopL_BeUSDA61** --HWVFCPHTASDAQINILRRAGLLPSNNSRTTSFTMLGMPHTAEFRQEGFVRIK**PS**MDA 247

GEC57378_BeNBRC14791 --HWVFCPHTASDAQINILRRAGLLPSNNSRTTSFTMLGMPHTAEFRQEGFVRIK**PS**MDA 230

WP_141713659_BeBLY3-8 --YWVFTGQTATAAQIDMLERSGVKPSKDHPTKVFTFLGVPHTAEWREEGFIRLT**PS**LDQ 256

WP_137479484_BeSemia938 --YWVFTGQTATDAEIDMLERSGVKPSREQPSKVFTLLGVPHTAEWRDEGFIRLT**PS**LDP 260

WP_155255696_BeWSM2783 --YWVFFPQTASNAQMNMLRSVGLMPSRDSPT-TFSIIGVPHTAEWRDEDFVRIN**PS**LDP 216

NopL_SfNGR234 --HWFLAPHMASEDQMSMLRATGLMPTAEVPTTTFLMMGMRHVAEFRGEGVIRIR**PS**VDF 336

CCE98803_SfHH103 --HWFLAPHMASEDQMSMLRATGLMPTAEVPTTTFLMMGMPHVAEFRGEGVIRIR**PS**LDF 336

WP_136510575_EspMPMI2T --HWVLAPHLASEDEMSMLRAKGLMPTAEVPTTTFLMMGMPHIAEFRGEGLIRIR**PS**LDL 345

WP_146207671_BspSUTN9-2 --DWVFTGQTATPAQIEMLRSRDLKPTRDVRTTTFTILGVPHTAEWREEGFIRLK**PS**LGP 188

SPP98400_BspORS3257 GLHWPNR--YACS--DRNAKKPQSHTDQGSQTITFTNLGVPVRAEWREEGSIRIK**PS**LNS 151

* * . : * :*: **:* *. :*: **:.

**NopL_BeUSDA61** GL----------------------- 249

GEC57378_BeNBRC14791 GL----------------------- 232

WP_141713659_BeBLY3-8 TLWPEEPAENRSLDSDSEEDPRIR- 280

WP_137479484_BeSemia938 TLWPEEPAEDRPADSEEDPPAR--- 282

WP_155255696_BeWSM2783 SLWPGYSGEDSPTG----------- 230

NopL_SfNGR234 DI----------------------- 338

CCE98803_SfHH103 DI----------------------- 338

WP_136510575_EspMPMI2T GI----------------------- 347

WP_146207671_BspSUTN9-2 SLWLEVRANTRQAAKHDDMKNAERS 213

SPP98400_BspORS3257 ALWPVGSSEHSPSG----------- 165

:

**Reference**

1. Ge, Y.-Y.; Xiang, Q.-W.; Wagner, C.; Zhang, D.; Xie, Z.-P.; Staehelin, C. The type 3 effector NopL of *Sinorhizobium* sp. strain NGR234 is a mitogen-activated protein kinase substrate. *J. Exp. Bot.* **2016**, *67*, 2483–2494, doi:10.1093/jxb/erw065.

2. Zhang, L.; Chen, X.-J.; Lu, H.-B.; Xie, Z.-P.; Staehelin, C. Functional analysis of the type 3 effector nodulation outer protein L (NopL) from *Rhizobium* sp. NGR234: symbiotic effects, phosphorylation, and interference with mitogen-activated protein kinase signaling. *J. Biol. Chem.* **2011**, *286*, 32178–32187, doi:10.1074/jbc.M111.265942.
