# Supplementary material for: Identification of Bradyrhizobium elkanii USDA61 Type III Effectors Determining Symbiosis with Vigna mungo
Source: Genes (Basel). 2020 Apr 27;11(5):474. doi: 10.3390/genes11050474 (PMC7291247; doi:10.3390/genes11050474)
Supplement: Supplementary file 1 [file genes-11-00474-s001.zip › Sup dataset_Nguyen et al_Genes 2020/FigS5_V. mungo morphology(ori).pptx]

## Slide 1
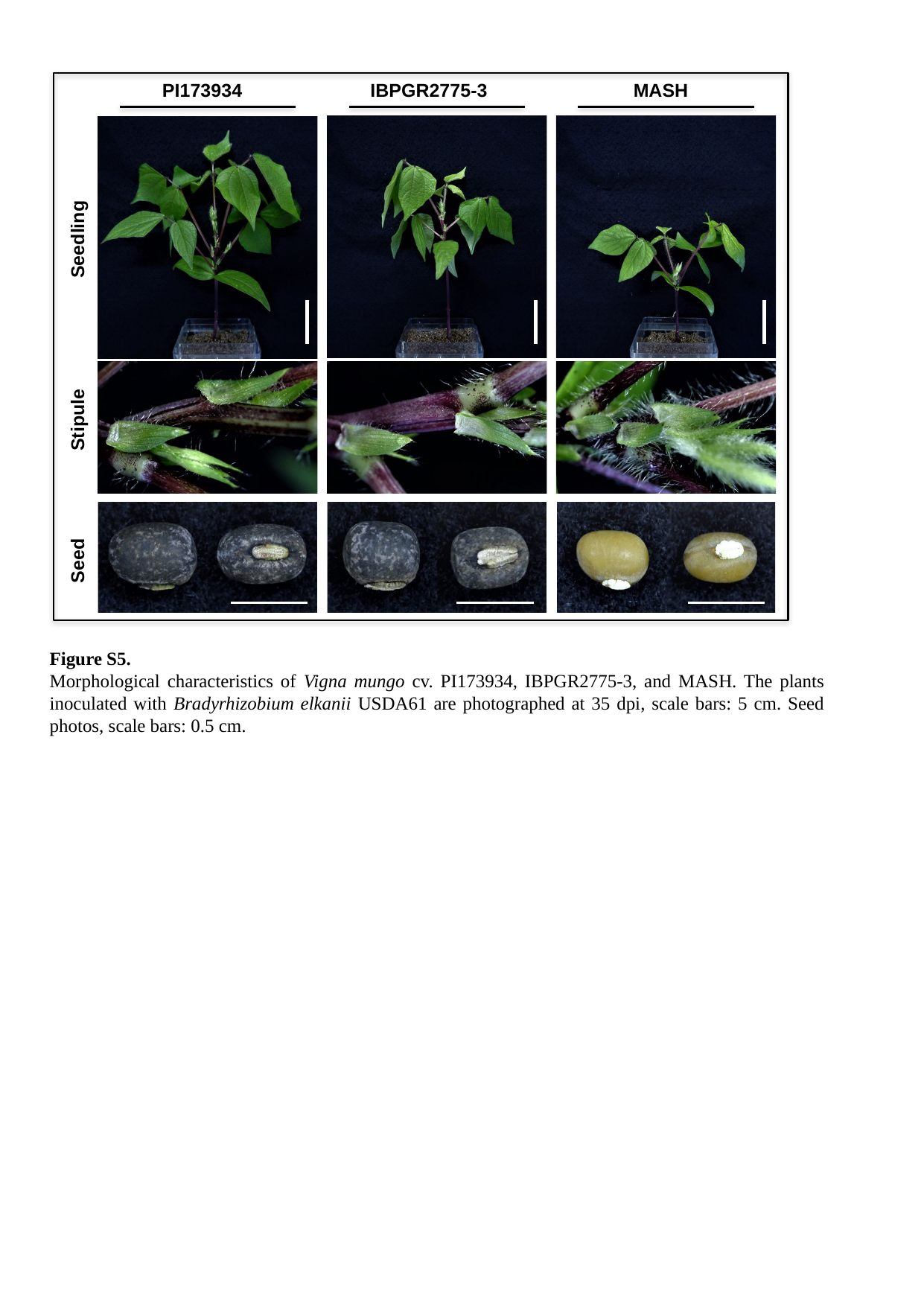

MASH
PI173934
IBPGR2775-3
Seedling
Stipule
Seed
Figure S5.
Morphological characteristics of Vigna mungo cv. PI173934, IBPGR2775-3, and MASH. The plants inoculated with Bradyrhizobium elkanii USDA61 are photographed at 35 dpi, scale bars: 5 cm. Seed photos, scale bars: 0.5 cm.
