# Supplementary material for: Identification of Bradyrhizobium elkanii USDA61 Type III Effectors Determining Symbiosis with Vigna mungo
Source: Genes (Basel). 2020 Apr 27;11(5):474. doi: 10.3390/genes11050474 (PMC7291247; doi:10.3390/genes11050474)
Supplement: Supplementary file 1 [file genes-11-00474-s001.zip › Sup dataset_Nguyen et al_Genes 2020/FigS3_PI sym properties(ori).pptx]

## Slide 1
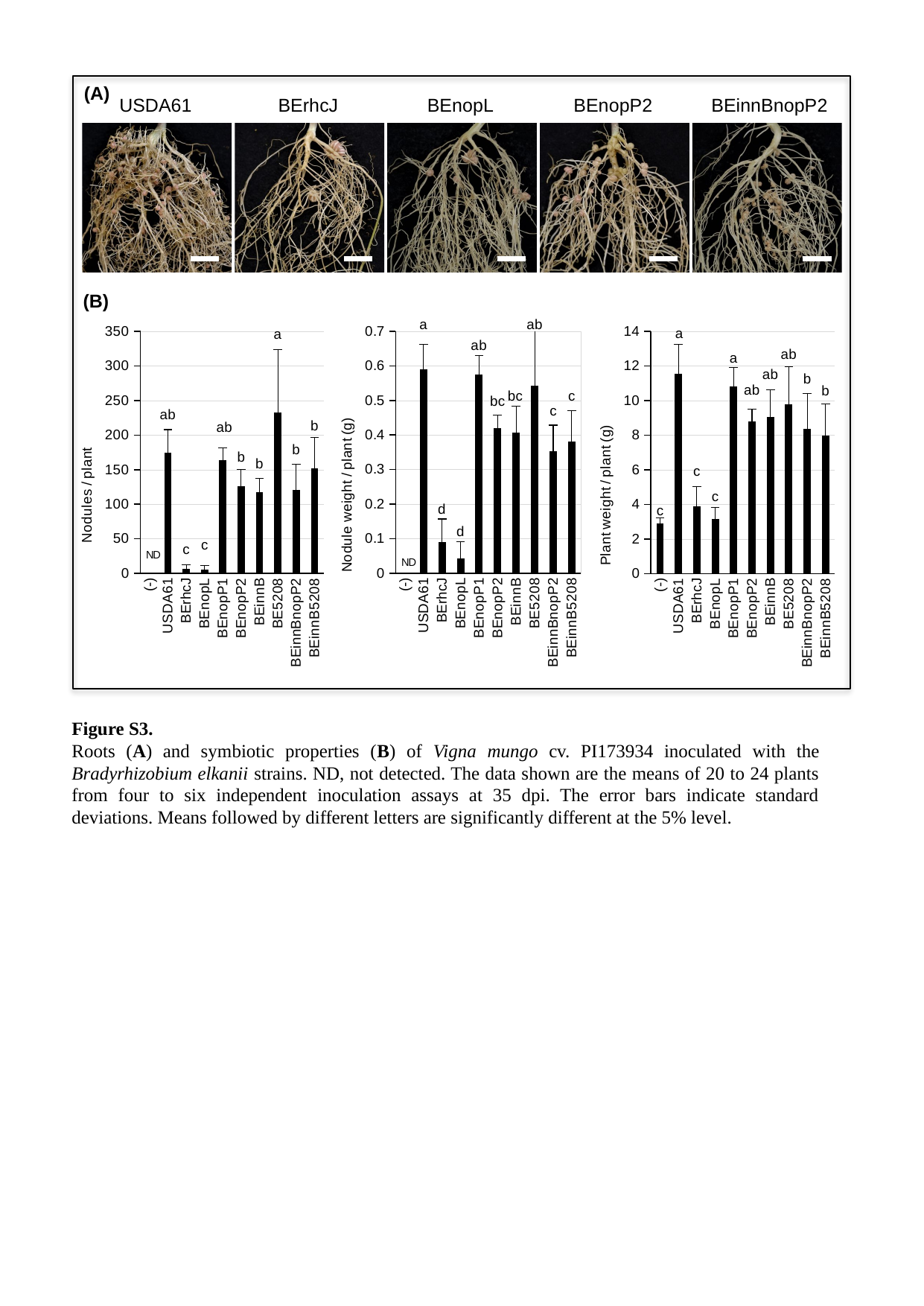

(A)
USDA61
BErhcJ
BEnopL
BEnopP2
BEinnBnopP2
(B)
### Chart
| Category | Large nodules | Small nodules |
|---|---|---|
| (-) | 0.0 | 0.0 |
| USDA61 | 174.2 | 0.0 |
| BErhcJ | 6.545454545454546 | 0.0 |
| BEnopL | 6.0 | 0.0 |
| BEnopP1 | 163.75 | None |
| BEnopP2 | 125.8 | 0.0 |
| BEinnB | 117.33333333333333 | 0.0 |
| BE5208 | 232.2 | 0.0 |
| BEinnBnopP2 | 121.22222222222223 | 0.0 |
| BEinnB5208 | 152.33333333333334 | 0.0 |
### Chart
| Category | Nodule weight (g) |
|---|---|
| (-) | 0.0 |
| USDA61 | 0.5894 |
| BErhcJ | 0.09054545454545455 |
| BEnopL | 0.04342105263157895 |
| BEnopP1 | 0.5752499999999999 |
| BEnopP2 | 0.42000000000000004 |
| BEinnB | 0.408 |
| BE5208 | 0.542 |
| BEinnBnopP2 | 0.35244444444444445 |
| BEinnB5208 | 0.3818333333333333 |
### Chart
| Category | Plant weight (g) |
|---|---|
| (-) | 2.8783333333333334 |
| USDA61 | 11.540000000000001 |
| BErhcJ | 3.8654545454545453 |
| BEnopL | 3.159473684210527 |
| BEnopP1 | 10.795 |
| BEnopP2 | 8.784 |
| BEinnB | 9.03 |
| BE5208 | 9.774000000000001 |
| BEinnBnopP2 | 8.35222222222222 |
| BEinnB5208 | 7.966666666666666 |Figure S3.
Roots (A) and symbiotic properties (B) of Vigna mungo cv. PI173934 inoculated with the Bradyrhizobium elkanii strains. ND, not detected. The data shown are the means of 20 to 24 plants from four to six independent inoculation assays at 35 dpi. The error bars indicate standard deviations. Means followed by different letters are significantly different at the 5% level.
