# Supplementary material for: Identification of Bradyrhizobium elkanii USDA61 Type III Effectors Determining Symbiosis with Vigna mungo
Source: Genes (Basel). 2020 Apr 27;11(5):474. doi: 10.3390/genes11050474 (PMC7291247; doi:10.3390/genes11050474)
Supplement: Supplementary file 1 [file genes-11-00474-s001.zip › Sup dataset_Nguyen et al_Genes 2020/FigS2_V. mungo cultivar sym(ori).pptx]

## Slide 1
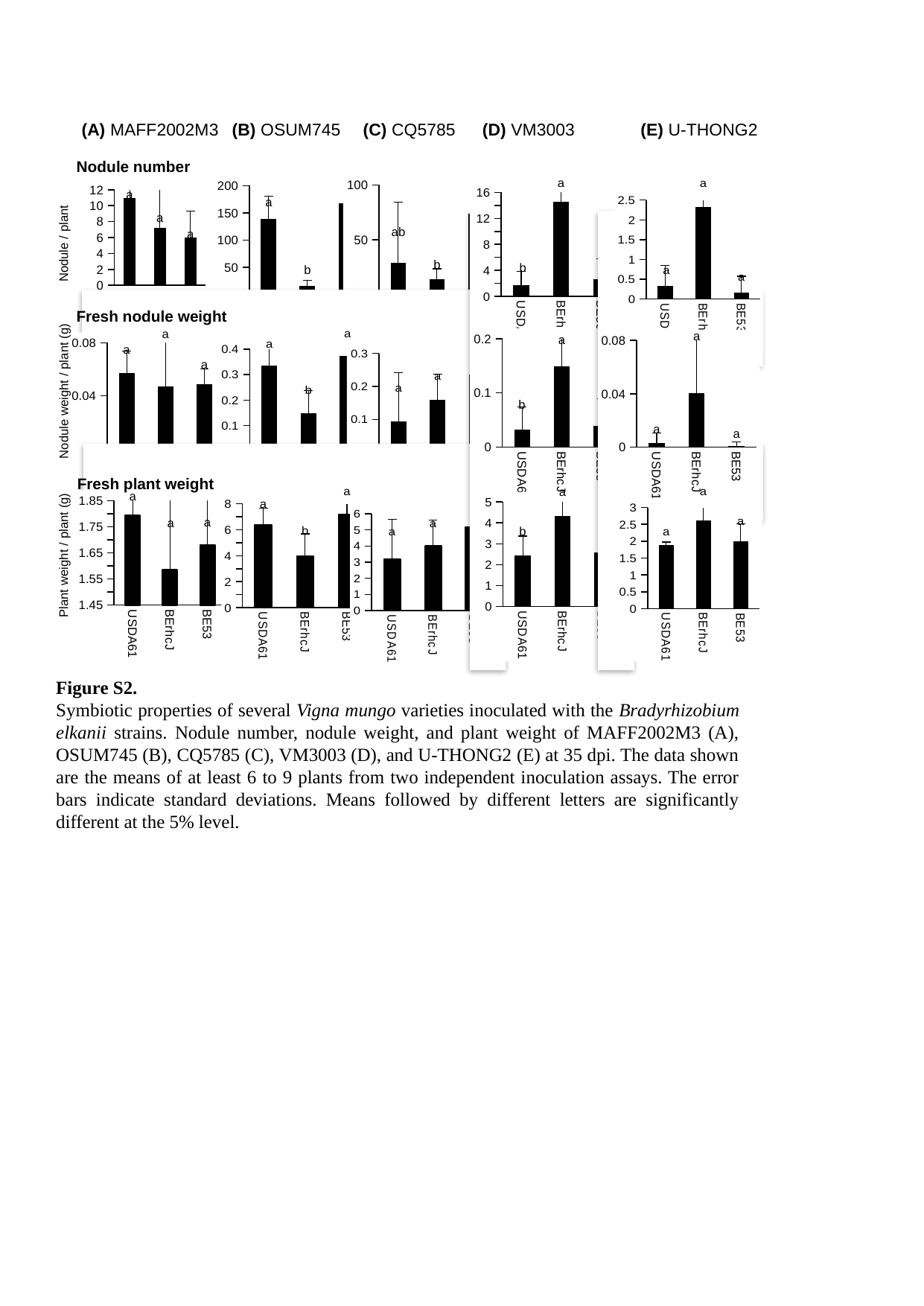

(A) MAFF2002M3
(B) OSUM745
(C) CQ5785
(D) VM3003
(E) U-THONG2
Nodule number
### Chart
| Category | Large nodules | Small nodules |
|---|---|---|
| USDA61 | 0.0 | 11.0 |
| BErhcJ | 0.0 | 7.25 |
| BE53 | 0.0 | 6.0 |
### Chart
| Category | Large nodules | Small nodules |
|---|---|---|
| USDA61 | 139.71428571428572 | 0.0 |
| BErhcJ | 16.375 | 0.0 |
| BE53 | 167.75 | 0.0 |
### Chart
| Category | Large nodules | Small nodules |
|---|---|---|
| USDA61 | 28.714285714285715 | 0.0 |
| BErhcJ | 14.0 | 0.0 |
| BE53 | 73.77777777777777 | 0.0 |
| BEnopL | 39.2 | 0.0 |
### Chart
| Category | Large nodules | Small nodules |
|---|---|---|
| USDA61 | 1.75 | 0.0 |
| BErhcJ | 14.5 | 0.0 |
| BE53 | 2.6 | 0.0 |
| BEnopL | 1.5 | 0.0 |
### Chart
| Category | Large nodules | Small nodules |
|---|---|---|
| USDA61 | 0.3333333333333333 | 0.0 |
| BErhcJ | 2.3333333333333335 | 0.0 |
| BE53 | 0.16666666666666666 | 0.0 |
Nodule / plant
Fresh nodule weight
### Chart
| Category | Nodule weight (g) |
|---|---|
| USDA61 | 0.3345714285714286 |
| BErhcJ | 0.14825 |
| BE53 | 0.374375 |
### Chart
| Category | Nodule weight (g) |
|---|---|
| USDA61 | 0.09385714285714286 |
| BErhcJ | 0.159 |
| BE53 | 0.23622222222222222 |
| BEnopL | 0.1478 |
### Chart
| Category | Nodule weight (g) |
|---|---|
| USDA61 | 0.05725 |
| BErhcJ | 0.04675 |
| BE53 | 0.04875 |
### Chart
| Category | Nodule weight (g) |
|---|---|
| USDA61 | 0.032 |
| BErhcJ | 0.1495 |
| BE53 | 0.03849999999999999 |
| BEnopL | 0.00925 |
### Chart
| Category | Nodule weight (g) |
|---|---|
| USDA61 | 0.00335 |
| BErhcJ | 0.04021666666666667 |
| BE53 | 0.0011666666666666668 |Nodule weight / plant (g)
Fresh plant weight
### Chart
| Category | Plant weight (g) |
|---|---|
| USDA61 | 6.397142857142856 |
| BErhcJ | 3.960000000000001 |
| BE53 | 7.158750000000001 |
### Chart
| Category | Plant weight (g) |
|---|---|
| USDA61 | 1.8929999999999998 |
| BErhcJ | 2.6206666666666667 |
| BE53 | 1.9953333333333336 |
### Chart
| Category | Plant weight (g) |
|---|---|
| USDA61 | 2.44375 |
| BErhcJ | 4.32 |
| BE53 | 2.5780000000000003 |
| BEnopL | 1.9699999999999998 |
### Chart
| Category | Plant weight (g) |
|---|---|
| USDA61 | 3.1757142857142857 |
| BErhcJ | 3.9928571428571433 |
| BE53 | 5.178888888888888 |
| BEnopL | 4.118 |
### Chart
| Category | Plant weight (g) |
|---|---|
| USDA61 | 1.79325 |
| BErhcJ | 1.58425 |
| BE53 | 1.6804999999999999 |Plant weight / plant (g)
Figure S2.
Symbiotic properties of several Vigna mungo varieties inoculated with the Bradyrhizobium elkanii strains. Nodule number, nodule weight, and plant weight of MAFF2002M3 (A), OSUM745 (B), CQ5785 (C), VM3003 (D), and U-THONG2 (E) at 35 dpi. The data shown are the means of at least 6 to 9 plants from two independent inoculation assays. The error bars indicate standard deviations. Means followed by different letters are significantly different at the 5% level.
