# Supplementary material for: Identification of Bradyrhizobium elkanii USDA61 Type III Effectors Determining Symbiosis with Vigna mungo
Source: Genes (Basel). 2020 Apr 27;11(5):474. doi: 10.3390/genes11050474 (PMC7291247; doi:10.3390/genes11050474)
Supplement: Supplementary file 1 [file genes-11-00474-s001.zip › Sup dataset_Nguyen et al_Genes 2020/FigS1_Vigna species sym(ori).pptx]

## Slide 1
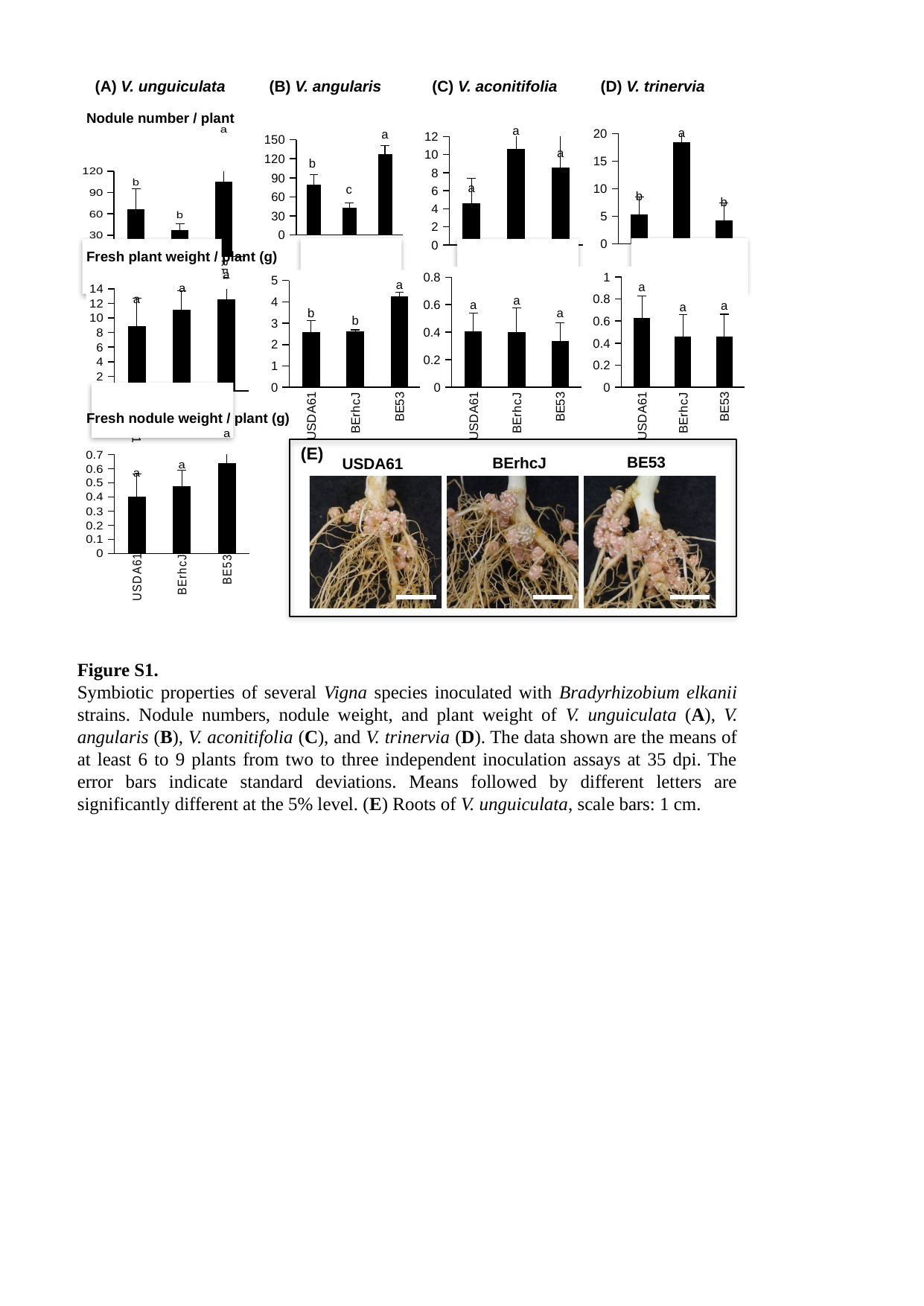

(A) V. unguiculata
(B) V. angularis
(C) V. aconitifolia
(D) V. trinervia
Nodule number / plant
### Chart
| Category | Large nodules | Small nodules |
|---|---|---|
| USDA61 | 78.66666666666667 | 0.0 |
| BErhcJ | 43.333333333333336 | 0.0 |
| BE53 | 127.66666666666667 | 0.0 |
### Chart
| Category | Large nodules | Small nodules |
|---|---|---|
| USDA61 | 5.333333333333333 | 0.0 |
| BErhcJ | 18.5 | 0.0 |
| BE53 | 4.333333333333333 | 0.0 |
### Chart
| Category | Large nodules | Small nodules |
|---|---|---|
| USDA61 | 4.6 | 0.0 |
| BErhcJ | 10.6 | 0.0 |
| BE53 | 8.6 | 0.0 |
### Chart
| Category | Large nodules | Small nodules |
|---|---|---|
| USDA61 | 66.5 | 0.0 |
| BErhcJ | 37.045 | 0.0 |
| BE53 | 105.0 | 0.0 |
Fresh plant weight / plant (g)
### Chart
| Category | Plant weight (g) |
|---|---|
| USDA61 | 0.63 |
| BErhcJ | 0.4575 |
| BE53 | 0.4566666666666667 |
### Chart
| Category | Plant weight (g) |
|---|---|
| USDA61 | 0.40499999999999997 |
| BErhcJ | 0.4029999999999999 |
| BE53 | 0.3348 |
### Chart
| Category | Plant weight (g) |
|---|---|
| USDA61 | 8.86975 |
| BErhcJ | 11.091500000000002 |
| BE53 | 12.564250000000001 |
### Chart
| Category | Plant weight (g) |
|---|---|
| USDA61 | 2.5766666666666667 |
| BErhcJ | 2.6166666666666667 |
| BE53 | 4.273333333333333 |
Fresh nodule weight / plant (g)
### Chart
| Category | Nodule weight (g) |
|---|---|
| USDA61 | 0.402 |
| BErhcJ | 0.478 |
| BE53 | 0.64025 |(E)
BE53
BErhcJ
USDA61
Figure S1.
Symbiotic properties of several Vigna species inoculated with Bradyrhizobium elkanii strains. Nodule numbers, nodule weight, and plant weight of V. unguiculata (A), V. angularis (B), V. aconitifolia (C), and V. trinervia (D). The data shown are the means of at least 6 to 9 plants from two to three independent inoculation assays at 35 dpi. The error bars indicate standard deviations. Means followed by different letters are significantly different at the 5% level. (E) Roots of V. unguiculata, scale bars: 1 cm.
