# Supplementary material for: Identification of Bradyrhizobium elkanii USDA61 Type III Effectors Determining Symbiosis with Vigna mungo
Source: Genes (Basel). 2020 Apr 27;11(5):474. doi: 10.3390/genes11050474 (PMC7291247; doi:10.3390/genes11050474)
Supplement: Supplementary file 1 [file genes-11-00474-s001.zip › Sup dataset_Nguyen et al_Genes 2020/TabS3_Seeds.docx]

**Table S3.** Legume seeds used in this study.

| **Plant seeds** | **Characteristics** | **Source (origin)^a^** |
| --- | --- | --- |
| ***V. mungo* (L.) Hepper** |  |  |
| cv. PI173934 | Wild-type | NARO (India) |
| cv. MASH | Wild-type | NARO (Nepal) |
| cv. COL/PAK/1991/IBPGR/2775(3) | Wild-type, designated as cv. IBPGR2775-3 in this study | NARO (Pakistan) |
| cv. COL/MYANMAR/2002/MAFF/2002M3 | Wild-type, designated as cv. MAFF2002M3 in this study | NARO (Myanmar) |
| cv. OSUM745 | Wild-type | NARO (Philippines) |
| cv. VM3003 | Wild-type | NARO (Thailand) |
| cv. U-THONG2 | Wild-type | NARO (Thailand) |
| cv. CQ5785 | Wild-type | NARO (Australia) |
|  |  |  |
| ***V. unguiculata* (L.) Walpers cv-gr. Sesquipedalis E. Westphal** |  |  |
| cv. COL/MYANMAR/2006/UTSUKUBA/078 | Wild-type | NARO (Myanmar) |
| ***V. aconitifolia* (Jacq.) Marechal** |  |  |
| cv. SSD4451-2008TN23 | Wild-type | NARO (India) |
| ***V. trinervia* (Heyne ex Wall.) Y. Tateishi & N. Maxted** |  |  |
| cv. COL/MYANMAR/2002/MAFF/2002M7 | Wild-type | NARO (Myanmar) |
| ***V. angularis* (Wild.) Ohwi et Ohashi** |  |  |
| cv. COL/TOYAMA/2015/NIAS/TOYAMA1-1 | Wild-type | NARO (Japan) |

^a^Japanese National Agriculture and Food Research Organization, Ibaraki, Japan.
