# Supplementary material for: Identification of Bradyrhizobium elkanii USDA61 Type III Effectors Determining Symbiosis with Vigna mungo
Source: Genes (Basel). 2020 Apr 27;11(5):474. doi: 10.3390/genes11050474 (PMC7291247; doi:10.3390/genes11050474)
Supplement: Supplementary file 1 [file genes-11-00474-s001.zip › Sup dataset_Nguyen et al_Genes 2020/FigS4_RhcJ&TtsI phylogenies(ori).pptx]

## Slide 1
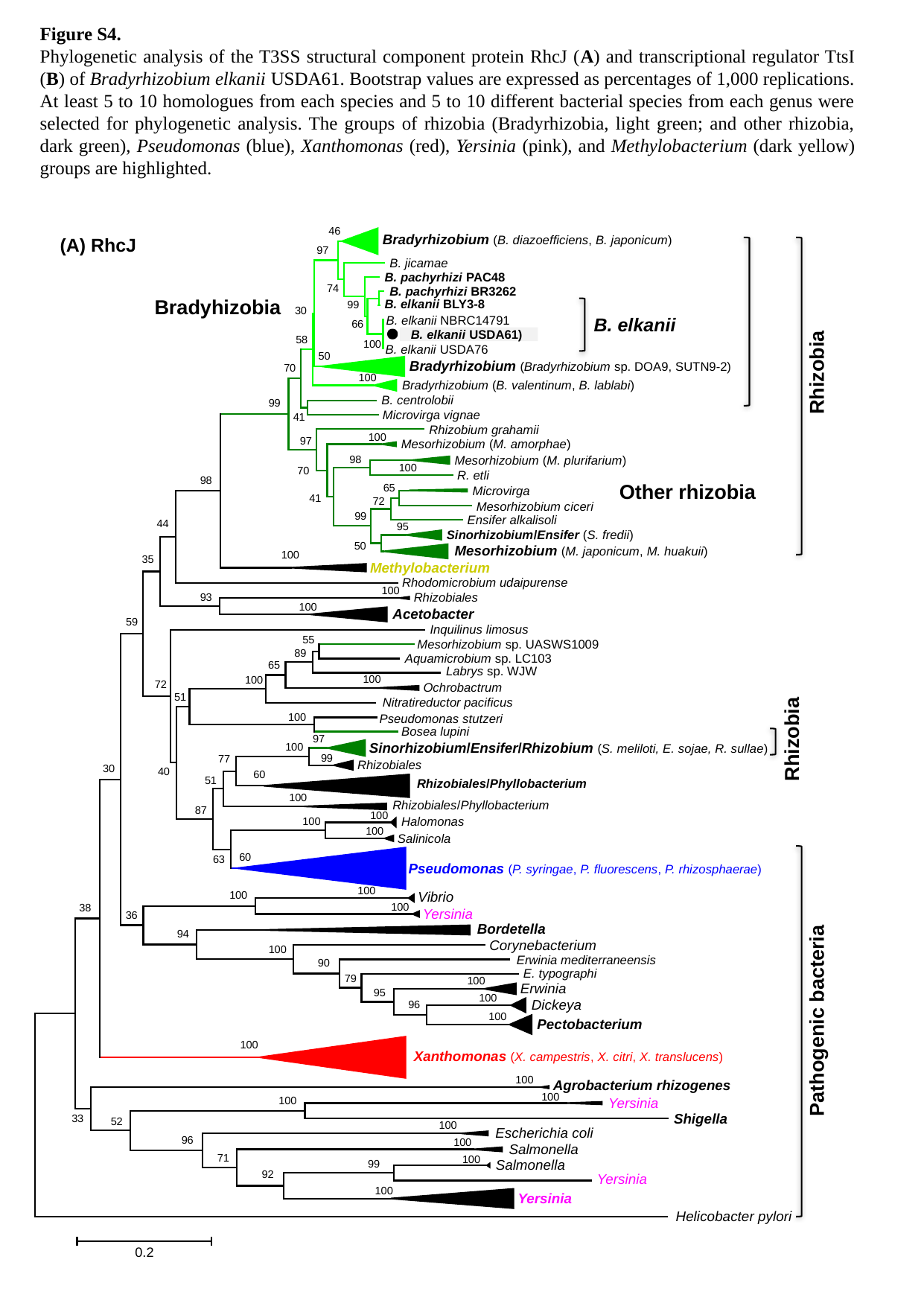

Figure S4.
Phylogenetic analysis of the T3SS structural component protein RhcJ (A) and transcriptional regulator TtsI (B) of Bradyrhizobium elkanii USDA61. Bootstrap values are expressed as percentages of 1,000 replications. At least 5 to 10 homologues from each species and 5 to 10 different bacterial species from each genus were selected for phylogenetic analysis. The groups of rhizobia (Bradyrhizobia, light green; and other rhizobia, dark green), Pseudomonas (blue), Xanthomonas (red), Yersinia (pink), and Methylobacterium (dark yellow) groups are highlighted.
Bradyrhizobium (B. diazoefficiens, B. japonicum)
B. jicamae
B. pachyrhizi PAC48
B. pachyrhizi BR3262
B. elkanii BLY3-8
B. elkanii NBRC14791
B. elkanii USDA61)
B. elkanii USDA76
Bradyrhizobium (Bradyrhizobium sp. DOA9, SUTN9-2)
Rhizobia
Bradyrhizobium (B. valentinum, B. lablabi)
B. centrolobii
Microvirga vignae
Rhizobium grahamii
Mesorhizobium (M. amorphae)
Mesorhizobium (M. plurifarium)
R. etli
Microvirga
Mesorhizobium ciceri
Ensifer alkalisoli
Sinorhizobium/Ensifer (S. fredii)
Mesorhizobium (M. japonicum, M. huakuii)
Methylobacterium
Rhodomicrobium udaipurense
Rhizobiales
Acetobacter
Inquilinus limosus
Mesorhizobium sp. UASWS1009
Aquamicrobium sp. LC103
Labrys sp. WJW
Ochrobactrum
Nitratireductor pacificus
Pseudomonas stutzeri
Bosea lupini
Rhizobia
Sinorhizobium/Ensifer/Rhizobium (S. meliloti, E. sojae, R. sullae)
Rhizobiales
Rhizobiales/Phyllobacterium
Rhizobiales/Phyllobacterium
Halomonas
Salinicola
Pseudomonas (P. syringae, P. fluorescens, P. rhizosphaerae)
Vibrio
Yersinia
Bordetella
Corynebacterium
Erwinia mediterraneensis
E. typographi
Erwinia
Dickeya
Pectobacterium
Xanthomonas (X. campestris, X. citri, X. translucens)
Agrobacterium rhizogenes
Yersinia
Shigella
Escherichia coli
Salmonella
Salmonella
Yersinia
Yersinia
Helicobacter pylori
46
97
74
99
30
66
58
100
50
70
100
99
41
100
97
98
100
70
98
65
41
72
99
44
95
50
100
35
100
93
100
59
55
89
65
100
100
72
51
100
97
100
99
77
30
40
60
51
100
87
100
100
100
60
63
100
100
100
38
36
94
100
90
79
100
95
100
96
100
100
100
100
100
33
52
100
96
100
71
100
99
92
100
B. elkanii
Pathogenic bacteria
Bradyhizobia
Other rhizobia
(A) RhcJ

## Slide 2
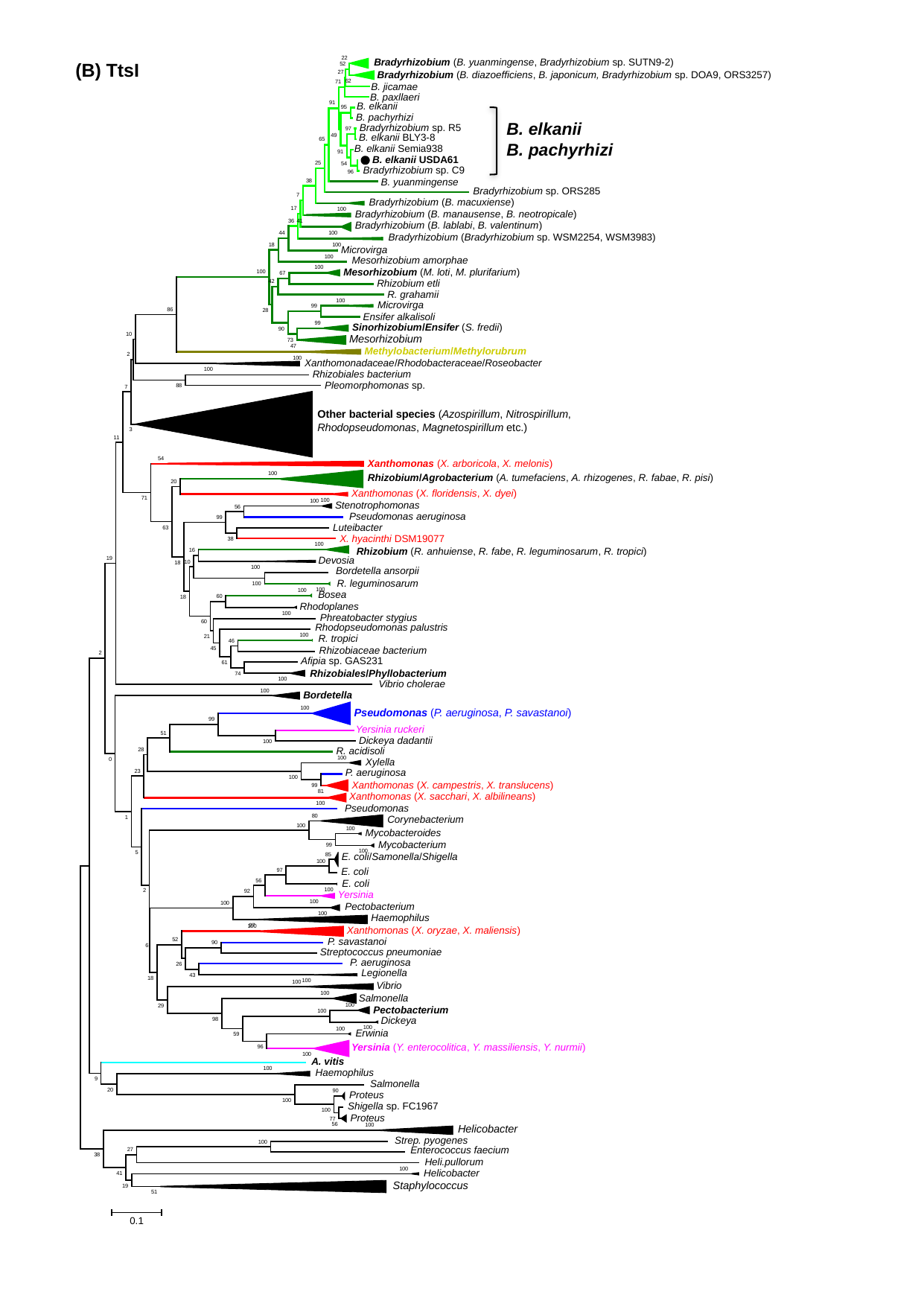

Bradyrhizobium (B. yuanmingense, Bradyrhizobium sp. SUTN9-2)
(B) TtsI
Bradyrhizobium (B. diazoefficiens, B. japonicum, Bradyrhizobium sp. DOA9, ORS3257)
B. jicamae
B. paxllaeri
B. elkanii
B. pachyrhizi
B. elkanii
B. pachyrhizi
Bradyrhizobium sp. R5
B. elkanii BLY3-8
B. elkanii Semia938
B. elkanii USDA61
Bradyrhizobium sp. C9
B. yuanmingense
Bradyrhizobium sp. ORS285
Bradyrhizobium (B. macuxiense)
Bradyrhizobium (B. manausense, B. neotropicale)
Bradyrhizobium (B. lablabi, B. valentinum)
Bradyrhizobium (Bradyrhizobium sp. WSM2254, WSM3983)
Microvirga
Mesorhizobium amorphae
Mesorhizobium (M. loti, M. plurifarium)
Rhizobium etli
R. grahamii
Microvirga
Ensifer alkalisoli
Sinorhizobium/Ensifer (S. fredii)
Mesorhizobium
Methylobacterium/Methylorubrum
Xanthomonadaceae/Rhodobacteraceae/Roseobacter
Rhizobiales bacterium
Pleomorphomonas sp.
Other bacterial species (Azospirillum, Nitrospirillum, Rhodopseudomonas, Magnetospirillum etc.)
Xanthomonas (X. arboricola, X. melonis)
Rhizobium/Agrobacterium (A. tumefaciens, A. rhizogenes, R. fabae, R. pisi)
Xanthomonas (X. floridensis, X. dyei)
Stenotrophomonas
Pseudomonas aeruginosa
Luteibacter
X. hyacinthi DSM19077
Rhizobium (R. anhuiense, R. fabe, R. leguminosarum, R. tropici)
Devosia
Bordetella ansorpii
R. leguminosarum
Bosea
Rhodoplanes
Phreatobacter stygius
Rhodopseudomonas palustris
R. tropici
Rhizobiaceae bacterium
Afipia sp. GAS231
Rhizobiales/Phyllobacterium
Vibrio cholerae
Bordetella
Pseudomonas (P. aeruginosa, P. savastanoi)
Yersinia ruckeri
Dickeya dadantii
R. acidisoli
Xylella
P. aeruginosa
Xanthomonas (X. campestris, X. translucens)
Xanthomonas (X. sacchari, X. albilineans)
Pseudomonas
Corynebacterium
Mycobacteroides
Mycobacterium
E. coli/Samonella/Shigella
E. coli
E. coli
Yersinia
Pectobacterium
Haemophilus
Xanthomonas (X. oryzae, X. maliensis)
P. savastanoi
Streptococcus pneumoniae
P. aeruginosa
Legionella
Vibrio
Salmonella
Pectobacterium
Dickeya
Erwinia
Yersinia (Y. enterocolitica, Y. massiliensis, Y. nurmii)
A. vitis
Haemophilus
Salmonella
Proteus
Shigella sp. FC1967
Proteus
Helicobacter
Strep. pyogenes
Enterococcus faecium
Heli.pullorum
Helicobacter
Staphylococcus
