# Supplementary material for: Identification of Bradyrhizobium elkanii USDA61 Type III Effectors Determining Symbiosis with Vigna mungo
Source: Genes (Basel). 2020 Apr 27;11(5):474. doi: 10.3390/genes11050474 (PMC7291247; doi:10.3390/genes11050474)
Supplement: Supplementary file 1 [file genes-11-00474-s001.zip › Sup dataset_Nguyen et al_Genes 2020/DataS3_NLS search(ori).docx]

**Data S3.**

Predictions of nuclear localization signals (NLSs) among rhizobial NopLs. (**A**) Bipartite motifs sequences of NLSs found in NopL of *B. elkanii* USDA61, *B. diazoefficiens* USDA110 (accession number BAC47075), *Sinorhizobium fredii* NGR234 (accession number AAB91935), and USDA257 (accession number AAL98685). NLSs were predicted using the cNLS Mapper software [1]. No NLS was found in NopLs of USDA110 and USDA257. (**B**–**E**) Locations of the NLSs and Serine-Proline (SP) motifs in the amino acid sequences. The bipartite motifs of NLSs (yellow) and SP motifs (blue) highlighted.

**(A)**

| Strains/NopLs | Sequences | Score |
| --- | --- | --- |
| USDA61/NopL | EPNFQRRSRMDFNSI**SP**TNT**SP**QPD**SP**SAPAGP | 2.6 |
|  |  |  |
| NGR234/NopL | DLETKHPYSQYLDWANPSLLDWQQDLHTRAT | 3.8 |
|  |  |  |
| USDA257/NopL | Not found |  |
|  |  |  |
| USDA110/NopL | Not found |  |

**(B)** USDA61 NopL (249 residues)

MAFVELWLEPNFQRRSRMDFNSI**SP**TNT**SP**QPD**SP**SAPAGPAGFEHQLREVEDSALPPAAG**SP**VQQGKAY**SP**YLDARHPYSQYLESGHPYSSLLDREDDLYAPAAP**SP**GPLVAARES**SP**QPGSQQPIAQAIAELPEFDPDLIWQNVEAGSSQAGPSQAGPSQAGPSSSAGAALSELTNFIPEDERFIADHWVFCPHTASDAQINILRRAGLLPSNNSRTTSFTMLGMPHTAEFRQEGFVRIKPSMDAGL

**(C)** NGR234 NopL (338 residues)

MDINST**SP**LNA**SP**QPD**SP**PPANASAFAHQLSGFQY**SP**PHAADSLLPQVEAD**SP**YLDTRHPYSQYLDSAYPYP**SP**CEWQHDLYTRTRER**SP**HPSEQRPHARVLQGAPEHDQDQHLEAAGPREGSWQVGPSRSGPSQAGL**SP**SATPLNP**SP**PPHATDLETKHPYSQYLDWANPSLLDWQQDLHTRATA**SP**APLTAERGR**SP**QPSEQQPHARALQVPEYDQDLIWQRVDAAGPQAGPWQVGPSHSGPSQARPSHAWPSSSAGAEPAELSDFVMDSGVRAWDHWFLAPHMASEDQMSMLRATGLMPTAEVPTTTFLMMGMRHVAEFRGEGVIRIRPSVDFDI

**(D)** USDA257 NopL (338 residues)

MDINSTRPLNA**SP**QPD**SP**PPANESAFAHQLSGFQY**SP**PHAADSLLPQVEAD**SP**YLDTGHPYSQYLDSAYPYP**SP**CEWQHDLYTRTRER**SP**HPSEQRPHARVLQDAPEHDQDQHVEAAGPRAGSWQVGPSRSGPSQAGPSSSATPLNA**SP**PPHATDLETEHPYSQYLDWANPSLLDWQHDLHTRATA**SP**APLTAERGK**SP**QPSEQQPHARALQVPEYDQDLIWQRVDAAGPQAGPWQVGPSHSGPSQARPSHAWPSSSAGAEPTELSDFVMDSGVRAWDHWFLAPHMASEDQMSMLRATGLMPTAEVPTTTFLMMGMPHVAEFRGEGVIRIRPSLDFDI

**(E)** USDA110 NopL (167 residues)

MDFNAVAPANT**SP**EPDTARTATDATEFERQLSGSEAPASAQGVAHPVLQGEAY**SP**YLDAGHPY**SP**YLETGHLYPPYPDLAHPLGPDSGWQDNLYAAPAAVAAPEPDNGQQHL**SP**QAIAQAIEEHPGFDQDVIWQTLDVGPSEAEPRHGEPQAGTSHAGPSRTAPFSG

**Reference**

1. Kosugi, S.; Hasebe, M.; Tomita, M.; Yanagawa, H. Systematic identification of cell cycle-dependent yeast nucleocytoplasmic shuttling proteins by prediction of composite motifs. *Proc. Natl. Acad. Sci. U. S. A.* **2009**, *106*, 10171–10176, doi:10.1073/pnas.0900604106.
