# Supplementary material for: Identification of Bradyrhizobium elkanii USDA61 Type III Effectors Determining Symbiosis with Vigna mungo
Source: Genes (Basel). 2020 Apr 27;11(5):474. doi: 10.3390/genes11050474 (PMC7291247; doi:10.3390/genes11050474)
Supplement: Supplementary file 1 [file genes-11-00474-s001.zip › Sup dataset_Nguyen et al_Genes 2020/DataS2_Align NopPs vs. NopP peptides.docx]

**Data S2.**

Alignment of the NopP1, NopP2, and NopP peptide fragments previously detected in the extracellular proteins of *B. elkanii* USDA61. The conserved residues among NopP peptide fragments (designated F1 to F3) and USDA61-type NopP2, and *B. diazoefficiens* USDA110-type NopP (accession number BAC47017) are highlighted in black. The residues of USDA61-type NopP1 differing from others are highlighted in grey.

NopP1_BeUSDA61 MYGRIGGYYEAVTWASHDEHADDRDFEGRFANMHLSAAEPTSSSAAPTYSLVTKPPIEPI 60

**NopP2_BeUSDA61** MYGRIVGSSSPSTGASQADEAGEAGDSSHFTEMVA--GVGSSGASPARYSLESNPPISEI 58

BAC47017/Blr1752_BdUSDA110 ----------------------------------M--EPGGSSSETRAYYLNSGPPIVEI 24

NopP_F1_BeUSDA61 ------------------------------------------------------------ 0

NopP_F2_BeUSDA61 ------------------------------------------------------------ 0

NopP_F3_BeUSDA61 ------------------------------------------------------------ 0

NopP1_BeUSDA61 DKDTFRREAKIFQNDHEIMRIAENPREYSRFVSTRAKNVREAAEDYGSTR--DSEEARYY 118

**NopP2_BeUSDA61** DRSSFNDGLWRFL-GSDIQSIASSREEYSDFVSKKAERAATVAGSYLHTYDDLSRPAKFF 117

BAC47017/Blr1752_BdUSDA110 DQLSFDRGLQRFL-GRDIMNIAINPQEYSDFVSKKAERAATVAGSYSATHYDPARPVRFF 83

NopP_F1_BeUSDA61 ------------------------------------------------------------ 0

NopP_F2_BeUSDA61 ------------------------------------------------------------ 0

NopP_F3_BeUSDA61 ------------------------------------------------------------ 0

NopP1_BeUSDA61 SYNLGNKTVALLRTEGGYSMNEFHDDRWRELFPGR**EHITSVVDLQ**LAHPLVENAGDILLE 178

**NopP2_BeUSDA61** SYKLGDETVGLLRVGGPVRIK---GDAFRNQ-FGR**NDLTSVVDLR**VTHPLVENAGDILLE 173

BAC47017/Blr1752_BdUSDA110 SYQLGDETVGLLRAGGPVRIK---GETFREK-FGR**NDLTSVVDLR**VTHPLVENAGDILLE 139

NopP_F1_BeUSDA61 -----------------------------------**NDLTSVVDLR**--------------- 10

NopP_F2_BeUSDA61 ------------------------------------------------------------ 0

NopP_F3_BeUSDA61 ------------------------------------------------------------ 0

NopP1_BeUSDA61 YQLRRDAREGEQPLLKWYPLNEESKAR**AAKLGFVEVDDCNMVLDPTQHPDKWTT**NSAGEW 238

**NopP2_BeUSDA61** HQLRIDARNGAEPLILSKPALGGMEPR**LAEMGFVHVGRNHWVLDPHQHPEVWTK**NENDEW 233

BAC47017/Blr1752_BdUSDA110 YQLRE---DGDDPLILSKPGLPGMEPR**LAEMGFVHVGRNHWVLDPHQHPEVWTK**NENDQW 196

NopP_F1_BeUSDA61 ------------------------------------------------------------ 10

NopP_F2_BeUSDA61 ---------------------------**LAEMGFVHVGR**---------------------- 11

NopP_F3_BeUSDA61 --------------------------------------**NHWVLDPHQHPEVWTK**------ 16

NopP1_BeUSDA61 QRANKPERYLAKVDDGERRNTHVASSG---------YAYEDDFM------ 273

**NopP2_BeUSDA61** QRVGKPTKYLAKAGDGDSATQAPRQFDSSDEDDSTEYYYLERALAGLHTE 283

BAC47017/Blr1752_BdUSDA110 QRVGKPTKYLSKVEDDDAAAESTVQADYSDEDDPS--VYLERVLTGLSME 244

NopP_F1_BeUSDA61 -------------------------------------------------- 10

NopP_F2_BeUSDA61 -------------------------------------------------- 11

NopP_F3_BeUSDA61 -------------------------------------------------- 16
