# Supplementary material for: Identification of Bradyrhizobium elkanii USDA61 Type III Effectors Determining Symbiosis with Vigna mungo
Source: Genes (Basel). 2020 Apr 27;11(5):474. doi: 10.3390/genes11050474 (PMC7291247; doi:10.3390/genes11050474)
Supplement: Supplementary file 1 [file genes-11-00474-s001.zip › Sup dataset_Nguyen et al_Genes 2020/TabS2_Oligonucleotides.docx]

**Table S2.** DNA oligonucleotide primers used in this study.

| **Oligonucleotides** | **Sequences** | **Usage** |
| --- | --- | --- |
| BeInnB-dcF1 | 5’-ACATGATTACGAATTCGCTCAACCCCTTTTGGTCTC-3’ | Construction of *innB* deletion |
| BeInnB-dcR1 | 5’-CTGCGCTCTCTGCAGCCTGCTCTCCTCGTGATGG-3’ |  |
| BeInnB-dcF2 | 5’-GAGAGCAGGCTGCAGAGAGCGCAGGCGGACGGC-3’ |  |
| BeInnB-dcR2 | 5’-GGCCAGTGCCAAGCTTGGCGCCCGAGAAGACACCAC-3’ |  |
| BeNopL-dcF1 | 5’-ACATGATTACGAATTCCTGATCGATTGGGCCTTAAA-3’ | Construction of *nopL* deletion |
| BeNopL-dcR1 | 5’-GTCCAGAGGATCCTTGCATTGCGACCTCCAGAG-3’ |  |
| BeNopL-dcF2 | 5’-AATGCAAGGATCCTCTGGACAATTACACGGCGA-3’ |  |
| BeNopL-dcR2 | 5’-GGCCAGTGCCAAGCTTAGCTTCGATGCGTTCAAGAC-3’ |  |
| BeNopP2-dcF1 | 5’-ACATGATTACGAATTCATGATGCCTATGCCGAACTC-3’ | Construction of *nopP2* deletion |
| BeNopP2-dcR1 | 5’-AGCTGCTGGATCCCAGATCGCTCTCCTATCGGC-3’ |  |
| BeNopP2-dcF2 | 5’-CGATCTGGGATCCAGCAGCTGTAAGTCGCGGGA-3’ |  |
| BeNopP2-dcR2 | 5’-GGCCAGTGCCAAGCTTCCGTATGGATCGAGGAACTC-3’ |  |
| Be5208-dcF1 | 5’-ACATGATTACGAATTCGCCTATGGTGTCGTTTGCTT-3’ | Construction of *bel2-5* deletion |
| Be5208-dcR1 | 5’-GGCGACATCTAGACCGTTCTCACCTTCAAAATA-3’ |  |
| Be5208-dcF2 | 5’-AGAACGGTCTAGATGTCGCCGTGGGCGGATAGC-3’ |  |
| Be5208-dcR2 | 5’-GGCCAGTGCCAAGCTTAGCCGTTTTGGAAGAAACCT-3’ |  |
| BeNopP1_F | 5’-ACCGCGGTGGCGGCCTATTCCCTCGTGACCAAGCC-3’ | Construction of *nopP1* single-crossover mutation |
| BeNopP1_R | 5’-CGGGGGATCCACTAGCGCTATTCGTTGTCCATTTG-3’ |  |
| aadAfor | 5’-TGATTTGCTGGTTACGGTGA-3’ | Amplification of the Streptomycin-resistant gene (*aadA*) in the pCAM120 plasmid for GUS tag verification |
| aadArev | 5’-TACTGCGCTGTACCAAATGC-3’ |  |
