# Supplementary material for: Identification of Bradyrhizobium elkanii USDA61 Type III Effectors Determining Symbiosis with Vigna mungo
Source: Genes (Basel). 2020 Apr 27;11(5):474. doi: 10.3390/genes11050474 (PMC7291247; doi:10.3390/genes11050474)
Supplement: Supplementary file 1 [file genes-11-00474-s001.zip › Sup dataset_Nguyen et al_Genes 2020/TabS1_Strains&plasmids_revised_HPN_SO.docx]

**Table S1.** Bacterial strains and plasmids used in this study.

| **Strains or plasmids** | **Characteristics^a^** | **References** |
| --- | --- | --- |
| **Bacterial strains** |  |  |
| ***Bradyrhizobium elkanii*** |  |  |
| USDA61 | Wild-type strain, Pol^r^ | USDA^b^ |
| BEnodC | USDA61 derivative harboring insertion in *nodC* gene, Pol^r^, Km^r^, Tc^r^ | [1] |
| BEttsInod | USDA61 derivative harboring insertion in *ttsI* and *nodC* genes, Pol^r^, Km^r^, Sm^r^, Tc^r^ | [1] |
| BErhcJ | USDA61 derivative harboring insertion in *rhcJ* encoding a membrane protein of the type III secretion apparatus, defective in type III protein secretion, Pol^r^, Km^r^, Tc^r^ | [2] |
| BE53 | USDA61 derivative carrying a Tn*5* insertion in the *innB* gene, Pol^r^, Km^r^ | [3] |
| BEinnB | USDA61 derivative with the *innB* gene deleted via double-crossover, Pol^r^ | This study |
| BE2-5 | USDA61 derivative with the *bel2-5* gene deleted via double-crossover, Pol^r^ | This study |
| BEnopL | USDA61 derivative with the *nopL* gene deleted via double-crossover, Pol^r^ | This study |
| BEnopP1 | USDA61 derivative harboring insertion of the plasmid pSUPSCAKm::*nopP1* in the *nopP1* gene via single-crossover, Pol^r^, Km^r^ | This study |
| BEnopP2 | USDA61 derivative with the *nopP2* gene deleted via double-crossover, Pol^r^ | This study |
| BEinnBnopP2 | USDA61 derivative with both *innB* and *nopP2* deleted via double-crossover, Pol^r^ | This study |
| BEinnB5208 | USDA61 derivative with both *innB* and *bel2-5* deleted via double-crossover, Pol^r^ | This study |
| USDA61G | USDA61 derivative containing a pCAM120 plasmid insertion, Pol^r^, Sm^r^/Sp^r^ | [4] |
| BEnodCG | BEnodC derivative containing a pCAM120 plasmid insertion, Tc^r^, Pol^r^, Km^r^, Sm^r^/Sp^r^ | This study |
| BEnopLG | BEnopL derivative containing a pCAM120 plasmid insertion, Pol^r^, Sm^r^/Sp^r^ | This study |
| ***Escherichia coli*** |  |  |
| HB101 | *recA*, *hsdR*, *hsdM*, *pro*, Sm^r^ | Invitrogen |
| DH5α | *sup*E44 ∆*lac*U169 (φ80 *lac*Z∆M15) *hsdR*17 *recA*1 *endA1* *gyrA*96 *thi*-1 *rel*A1 | BRL, Bethesda, MD, U.S.A. |
| S17-1 | *thi pro hsd*R^-^ *hsd*M^+^ *recA* RP4::2-Tc::Mu-Km::Tn*7*(Tp^r^/Sm^r^) | [5] |
| **Plasmids** |  |  |
| pRK2013 | Helper plasmid, ColE1 replicon carrying RK2 transfer genes; Km^r^, tra | [6] |
| pCAM120 | mTn*5*SS*gusA20* (P*aph*-*gusA*-*trpA* ter translational fusion) in pUT/mini-Tn*5*; Sm^r^/Sp^r^, Ap^r^ | [7] |
| pSUPSCAKm | Derivative of pSUPPOL2SCA [8] with a kanamycin resistance gene in the  pSUPSCAKm  *Dra*I site, *oriT* of RP4, Tc^r^, Km^r^ | [3] |
| pSUPSCAKm::*nopP1* | pSUPSCAKm carrying a 0.5-kb DNA fragment containing the internal sequence of USDA61 *nopP1*, Km^r^ | This study |
| pK18mobsacB | Mobilizable vector for gene disruption and replacement, Mob+ *sacB*, Km^r^ | [9] |
| pInnBUD | pK18mobsacB containing insertions of upstream and downstream DNA fragments of the USDA61 *innB*, Km^r^ | This study |
| pBel2-5UD | pK18mobsacB containing insertions of upstream and downstream DNA fragments of the USDA61 *bel2-5*, Km^r^ | This study |
| pNopLUD | pK18mobsacB containing insertions of upstream and downstream DNA fragments of the USDA61 *nopL*, Km^r^ | This study |
| pNopP2UD | pK18mobsacB containing insertions of upstream and downstream DNA fragments of the USDA61 *nopP2*, Km^r^ | This study |

^a^Pol^r^, polymyxin resistant; Km^r^, kanamycin resistant; Sm^r^, streptomycin resistant; Sp^r^, spectinomycin resistant; Tc^r^, tetracycline resistant; Tp^r^, trimethoprim resistant; Ap^r^, ampicillin resistant.

^b^United States Department of Agriculture (USDA), Beltsville, MD.

**References**

1. Okazaki, S.; Kaneko, T.; Sato, S.; Saeki, K. Hijacking of leguminous nodulation signaling by the rhizobial type III secretion system. *Proc. Natl. Acad. Sci. U. S. A.* **2013**, *110*, 17131–17136, doi:10.1073/pnas.1302360110.

2. Okazaki, S.; Zehner, S.; Hempel, J.; Lang, K.; Göttfert, M. Genetic organization and functional analysis of the type III secretion system of *Bradyrhizobium elkanii*. *FEMS Microbiol. Lett.* **2009**, *295*, 88–95, doi:10.1111/j.1574-6968.2009.01593.x.

3. Nguyen, H. P.; Miwa, H.; Kaneko, T.; Sato, S.; Okazaki, S. Identification of *Bradyrhizobium elkanii* genes involved in incompatibility with *Vigna radiata*. *Genes (Basel).* **2017**, *8*, 374, doi:10.3390/genes8120374.

4. Nguyen, H. P.; Ratu, S. T. N.; Yasuda, M.; Göttfert, M.; Okazaki, S. InnB, a novel Type III effector of *Bradyrhizobium elkanii* USDA61, controls symbiosis with *Vigna* species. *Front. Microbiol.* **2018**, *9*, 3155, doi:10.3389/FMICB.2018.03155.

5. Simon, R.; Priefer, U.; Pühler, A. A broad host range mobilization system for *in vivo* genetic engineering: Transposon mutagenesis in Gram negative bacteria. *Bio/Technology* **1983**, *1*, 784–791, doi:10.1038/nbt1183-784.

6. Figurski, D. H.; Helinski, D. R. Replication of an origin-containing derivative of plasmid RK2 dependent on a plasmid function provided in *trans*. *Proc. Natl. Acad. Sci. U. S. A.* **1979**, *76*, 1648–1652.

7. Wilson, K. J.; Sessitsch, A.; Corbo, J. C.; Giller, K. E.; Akkermans, A. D. L.; Jefferson, R. A. *β*-Glucuronidase (GUS) transposons for ecological and genetic studies of rhizobia and other Gram-negative bacteria. *Microbiology* **1995**, *141*, 1691–1705.

8. Krause, A.; Doerfel, A.; Göttfert, M. Mutational and transcriptional analysis of the type III secretion system of *Bradyrhizobium japonicum*. *Mol. Plant-Microbe Interact.* **2002**, *15*, 1228–1235, doi:10.1094/MPMI.2002.15.12.1228.

9. Schafer, A.; Tauch, A.; Jager, W.; Kalinowski, J.; Thierbach, G.; Puhler, A. Small mobilizable multi-purpose cloning vectors derived from the *Escherichia coli* plasmids pK18 and pK19: selection of defined deletions in the chromosome of *Corynebacterium glutamicum*. *Gene* **1994**, *145*, 69–73, doi:10.1016/0378-1119(94)90324-7.
